# From Nature to Nanotech: Customizing Essential Oil Delivery for a Reduced Fungicide in Agriculture

**DOI:** 10.1021/acsomega.5c07651

**Published:** 2025-12-29

**Authors:** Michele Caroline Terra, Estefânia Vangelie Ramos Campos, Ana Cristina Preisler, Anderson do Espírito Santo Pereira, Jhones Luiz de Oliveira, Leonardo Fernandes Fraceto

**Affiliations:** † Institute of Science and Technology, 28108 São Paulo State University (UNESP), Av. Três de Março 511, 18087-180 Sorocaba, São Paulo, Brazil; ‡ B. Nano Soluções Tecnológicas Ltda, Avenida Itavuvu 11777, 18078-005 Sorocaba, São Paulo, Brazil

## Abstract

Modern agriculture is increasingly challenged by climate change, soil degradation, and the growing incidence of fungal diseases, which significantly reduce crop yields and quality. To mitigate these problems, the increased use of synthetic pesticides has become common; however, their overuse has led to environmental contamination, human health risks, and the emergence of resistant pathogens. Essential oils (EOs) have shown promise as a sustainable alternative due to their natural antifungal, antioxidant, and antimicrobial properties. Despite their potential, direct agricultural application of EOs is limited by volatility, poor water solubility, and instability under environmental conditions. Nanotechnology offers an innovative approach through the nanoencapsulation of EOs, enhancing their stability, bioavailability, and controlled release while minimizing volatilization losses. Among the available delivery systems, lipid-based nanoparticles, such as solid lipid nanoparticles (SLNs) and nanostructured lipid carriers (NLCs), stand out for their biocompatibility, environmental safety, and ability to prolong biological activity in the field. These systems also enable codelivery of EOs with biocontrol agents or micronutrients, thereby supporting integrated pest and disease management strategies. This review provides a timely and comprehensive synthesis of recent advances in the nanoencapsulation of EOs using lipid-based nanoplatforms, filling a critical gap in the literature by elucidating the pivotal role of NLCs in enabling sustainable, effective, and scalable strategies for fungal disease control in agriculture. Additionally, it highlights current challenges and future research directions related to large-scale production, field validation, and the assessment of ecological safety and potential effects on nontarget organisms.

## Graphical abstract

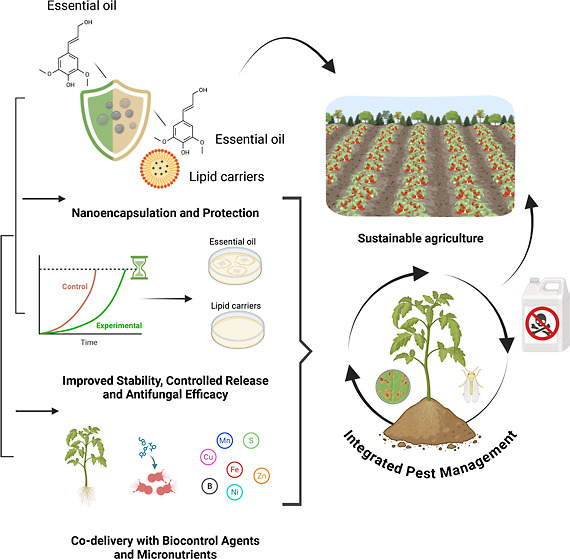

## 1 Introduction

Modern agriculture faces multiple challenges that threaten global food security and sustainability. Climate change, soil degradation, and water scarcity are key factors that compromise agricultural productivity.^1,60^ Rising temperatures and changes in precipitation patterns affect crop growth and influence the spread and severity of plant diseases. Among these, fungal diseases pose a significant threat, substantially reducing yield and quality.^2,3^ Phytopathogenic fungi cause severe agricultural losses, affecting food production and increasing economic risks for farmers. According to the FAO (2019), pests and diseases cause 20% to 40% losses in global agricultural production, resulting in estimated economic damages of USD 290 billion annually.^4^

To mitigate these losses, there has been a significant increase in the global use of synthetic pesticides, reaching approximately 3.69 million tons in 2022. However, excessive reliance on these chemicals has contributed to pathogen resistance, environmental contamination, and concerns for human health.^5^ In Brazil, soybean production, a cornerstone of the national economy, reached 166.1 million tons in the 2024/25 harvest.^6^ Like other crops, soybeans face persistent disease challenges that require effective control strategies. While synthetic fungicides offer rapid action and broad-spectrum effects, their overuse accelerates pathogen adaptation, reducing long-term efficacy and necessitating higher doses or frequent product replacements.^7^

Additionally, pesticide residues accumulate in soils and aquatic environments, impacting nontarget species and increasing environmental risks. According to Leoci,^8^ synthetic pesticide use in agriculture amounts to approximately 4.1 million tons per year, with more than 2000 active ingredients categorized into 60 chemical classes worldwide.^8^ Their widespread application has been linked to several adverse health effects, these include harms such as neurotoxic and endocrine effects, organ damage (liver and kidney), increased susceptibility to cancer, and reproductive disorders.^8^

Given these concerns, sustainable alternatives are being explored, and essential oils (EOs) have gained prominence as potential natural antifungal agents. Extracted from aromatic plants, these compounds exhibit antimicrobial, antifungal, and insect-repellent properties, making them a promising option for disease management in agriculture.^9^ Monoterpenes, such as citral, geraniol, eugenol, and thymol, have demonstrated vigorous antifungal activity and a favorable biodegradability profile, thereby reducing environmental impacts. However, their direct agricultural application is limited due to high volatility, limited stability, and susceptibility to environmental conditions such as light and temperature.

The application of essential oils in technical agricultural settings poses significant challenges regarding consistency and efficacy. Their properties can vary widely depending on agronomic factors and processing methods, undermining the standardization required for more demanding uses. Furthermore, the current production and supply infrastructure remains limited and poorly equipped to support large-scale demand, particularly in terms of sustainability criteria. In terms of performance, although essential oils have bioactive potential, many used in isolation still fall short of the effectiveness seen in conventional chemical inputs, particularly in biological control applications.^10,11^

To address these limitations, nanotechnology has emerged as a novel approach to improve the stability and sustained release of EOs through nanoencapsulation.^12^ Among the various technological platforms available, lipid-based nanoparticles such as solid lipid nanoparticles (SLNs) and NLCs, stand out as effective delivery systems for bioactive compounds. These nanosystems combine solid and liquid lipids, forming a flexible matrix that protects active ingredients from degradation, enhances their absorption by plants, and prolongs their biological activity.^13^

Additionally, the application of biodegradable substances, such as vegetable butters (coca, shea, and palm), natural waxes (beeswax), and vegetable oils (coconut), not only offers environmental benefits but also aligns with circular economy principles. Prioritizing byproducts of plant and animal origin for nanoparticle formulation reduces waste and promotes the efficient use of natural resources. Natural lipids provide advantages over synthetic and semisynthetic lipids, such as greater biocompatibility and lower in vivo toxicity, making them safer and more sustainable for agricultural applications.^14^ These systems also facilitate the controlled release of nutrients and agrochemicals, reducing chemical input use and minimizing environmental impacts.^15^

Thus, combining nanotechnology, lipid-based formulations, and essential oils represents an innovative approach to fungal control in agriculture. The development of versatile nanoplatforms can reduce reliance on synthetic pesticides and support more sustainable, efficient agricultural practices. This review aims to address the significant advances in the nanoencapsulation of essential oils, with a particular focus on lipid-based formulations, especially NLCs, for controlling agriculturally essential fungi.

## 2 Method for Literature Review

The literature search was conducted between January and September 2025 using the electronic databases Web of Science, Scopus, PubMed, ScienceDirect, and Google Scholar. The search aimed to identify peer-reviewed studies reporting the use of lipid-based nanocarriers—including solid lipid nanoparticles (SLNs), nanostructured lipid carriers (NLCs), and *nanoemulsions*—for the encapsulation of essential oils or their derivatives to control phytopathogenic fungi of agricultural relevance. The main keywords and boolean combinations used were: (“essential oil” OR “volatile compound” OR “terpene”) AND (“solid lipid nanoparticle” OR “nanostructured lipid carrier” OR “nanoemulsion” OR “lipid nanocarrier”) AND (“fungal control” OR “antifungal” OR “plant disease” OR “agriculture”). Studies were included if they (i) evaluated antifungal activity either in vitro, in vivo, or in planta; (ii) used a lipid-based nanosystem as the carrier matrix; and (iii) provided physicochemical characterization data (particle size, polydispersity index, zeta potential). Reviews, studies using nonlipidic carriers (e.g., polymeric nanoparticles, chitosan, silica), or those unrelated to agricultural pathogens were excluded.

## 3 Essential Oils as Antifungal Agents

Essential oils (EOs) exhibit various biological activities and serve as sustainable alternatives for controlling fungi in agriculture, offering significant advantages over synthetic fungicides, including biodegradability, lower environmental toxicity, and the absence of widespread fungal resistance. These plant-derived bioactive compounds, obtained from leaves, flowers, bark, and roots, are well-known for their fragrance and therapeutic potential. In agriculture, they have gained prominence as alternatives to conventional pesticides. Their antimicrobial, antifungal, antioxidant, and insect-repellent properties make them highly relevant for managing agricultural pests and diseases.^10^ EOs are not composed of a single compound but instead contain a wide variety of substances, with dozens or even hundreds of different compounds at varying concentrations. These include alcohols, terpenoids, esters, acids, epoxides, ketones, aldehydes, sulfides, and amines.^16,17^

The biological activities of secondary metabolites found in crude extracts, essential oils, or isolated active ingredients from various plant species can provide an effective method for alternative pest and disease control in food production. These alternatives result in higher quality, more functional foods while ensuring safer consumption by reducing health risks associated with fungicide residues and other synthetic substances.^9^

The antifungal activity of EOs is directly correlated to their chemical composition. Substances such as citral, eugenol, and isoeugenol exhibit antifungal activity in both liquid and vapor states, preventing mycelial germination and spore development of spores.^18^ For instance, eugenol can irreparably damage cell membranes, leading to cellular collapse. The phenolic components of EOs make the phospholipid bilayer of cell membranes more vulnerable, increasing permeability, causing the leakage of essential intracellular elements, and impairing microbial enzymatic systems.^19,20^

Various bioactive compounds in EOs exhibit potent antifungal activity through mechanisms such as membrane destabilization and rupture, enzymatic inhibition, generation of reactive oxygen species (ROS), and interference with fungal morphogenesis and reproduction. These different mechanisms are illustrated in Figure 1. Among the most studied compounds are thymol, eugenol, citral, carvacrol and cinnamaldehyde, each possessing specific characteristics that contribute to their antifungal action. In addition to these well-known molecules, other bioactive constituents, including linalool, geraniol, menthol, terpinen-4-ol, and anethole, have shown broad-spectrum antifungal effects, acting synergistically with major phenol compounds.

**Figure 1:**
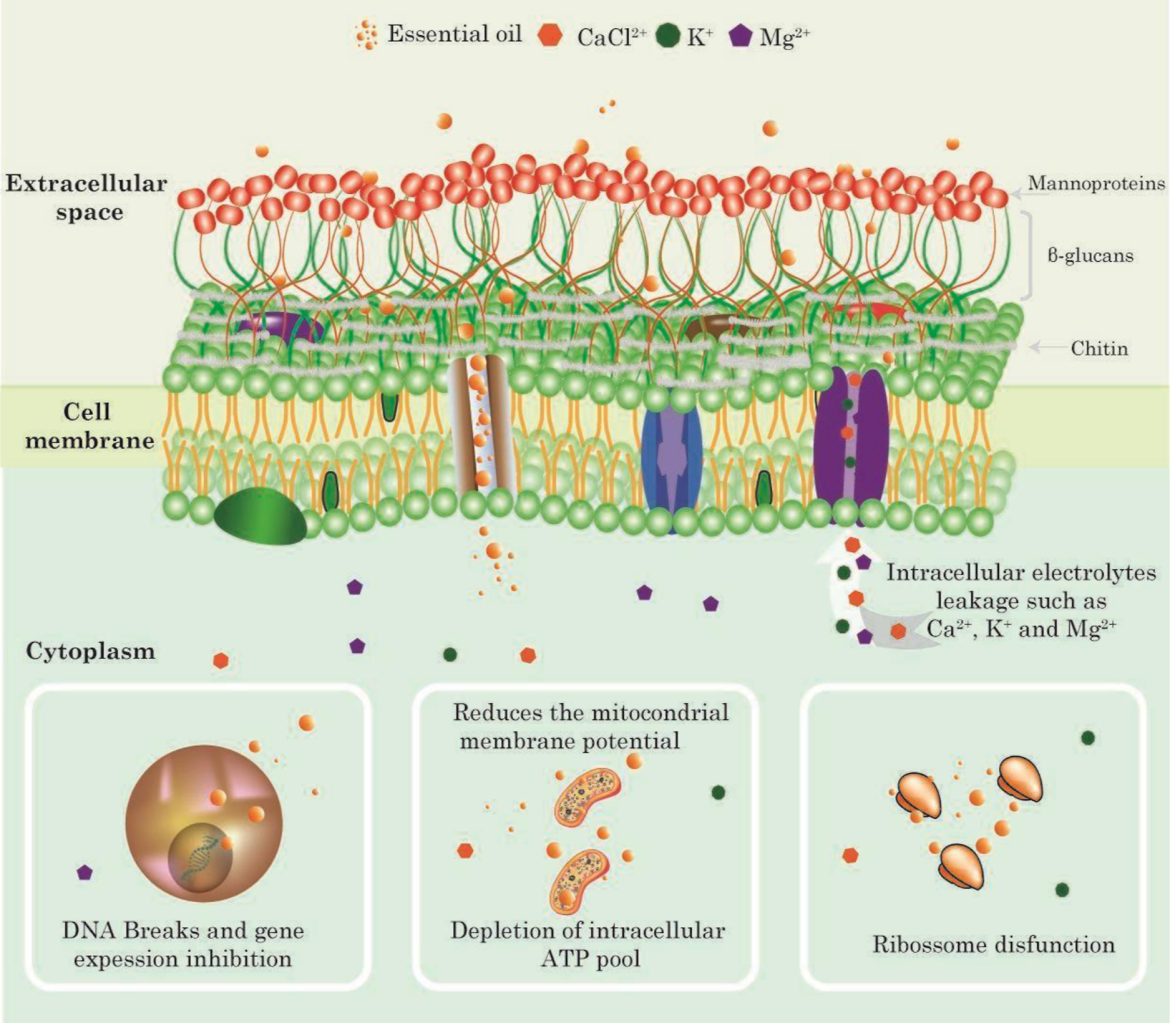
Mechanism of action of essential oils as fungicides. Once the cell absorbs, essential oils can cause DNA alterations or inhibit gene expression, induce mitochondrial changes, and disrupt ribosome function, ultimately leading to cell death.

The major components, their sources of EOs, and their mechanism of action are summarized in Table 1.

**Table 1:** Main Bioactive Components of Essential Oils with Antifungal Activity, the Oils in Which They are Found, Their Reported Mechanisms of Antifungal Action, and Corresponding References^a^

bioactive compound	essential oil	main antifungal mechanism of action	references
1,8—Cineole/eucalyptol	Eucalyptus oil, Rosemary oil, Cardamom oil	Affects cell membrane integrity and ion permeability in fungi. Can inhibit enzymes and essential metabolic pathways, impacting fungal proliferation	^21^
Alpha-pinene	Pine oil, Rosemary oil, Cypress oil	May interact with the fungal cell membrane, altering its structure and function. Has been associated with inhibition of hyphal growth and reduction of cell viability	^22^
β-Caryophyllene	Clove oil, Black Pepper oil, Rosemary oil, Lavender oil	Disrupts fungal cell membrane integrity, leading to increased permeability and leakage of cellular contents. Can also inhibit ergosterol biosynthesis and induce oxidative stress within fungal cells	^23,24^
Carvacrol	Oregano oil, Thyme oil	It causes the disintegration of the fungal cell membrane, leading to cell lysis and death. Affects ergosterol biosynthesis and inhibits hyphal and spore formation	^25,26^
Cinnamaldehyde	Cinnamon oil (bark)	Compromises fungal cell membrane integrity, inhibits ergosterol biosynthesis, affects the respiratory chain, and alters the activity of carbohydrate metabolism-related enzymes. Can inhibit mycelial growth and spore germination	^27,28^
Citral	Lemongrass oil, Litsea cubeba oil, Lemon verbena oil	Disrupts the fungal cell membrane structure and function, leading to increased permeability, leakage of intracellular components (e.g., K^+^ ions, proteins), and inhibition of metabolic enzymes. Can also inhibit spore germination and hyphal growth	^29^
Eugenol	Clove oil, Cinnamon oil (leaf), Basil oil	Increases fungal cell membrane permeability, causing leakage of intracellular constituents (ions, ATP, proteins), disrupting the electron transport chain, and inhibiting crucial metabolic enzymes. May also inhibit biofilm formation	^22,30^
Geraniol	Palmarosa oil, Geranium oil, Rose oil	Induces oxidative stress, damages the fungal cell membrane, affects ergosterol biosynthesis, and can cause DNA fragmentation, leading to programmed cell death	^31,32^
Limonene	Orange oil (Citrus sinensis), Lemon oil, Grapefruit oil	Disrupts the fungal cell membrane by interacting with lipid components, leading to increased permeability, loss of cellular contents, and inhibition of critical enzyme activities. Can also interfere with mitochondrial function and induce oxidative stress	^22^
Linalool	Lavender oil, Coriander oil, Basil oil	Increases fungal cell membrane permeability, resulting in the loss of ions and other intracellular components. Interferes with cell wall morphology and can inhibit biofilm formation	^33,34^
Menthol	Peppermint oil	Disrupts fungal cell membranes, interferes with energy metabolism, ion homeostasis, and can cause oxidative stress. Affects cellular morphology and inhibits mycelial growth	^35,36^
Pulegone	. Pennyroyal oil, Peppermint oil, Ziziphora clinopodioides oil	Disrupts fungal cell membranes, leading to loss of cellular integrity and leakage of intracellular components. Interferes with metabolic enzymes and can inhibit fungal growth and reproduction	^37,38^
Terpinen-4-ol	Tea Tree Oil	Causes loss of cell membrane integrity, leading to leakage of intracellular constituents. Inhibits spore germination and mycelial growth	^21^
Thymol	Thyme oil, Oregano oil	Disrupts the fungal cytoplasmic membrane, altering its fluidity and permeability, leading to the loss of ions (especially K^+^) and macromolecules. Inhibits ATPase activity and other energy-related cellular enzymes	^26,39^

a Most compounds exhibit multifactorial mechanisms, including disruption of cell membrane integrity, inhibition of key enzymes, alteration of ionic permeability, and interference with oxidative metabolism, which together contribute to their effectiveness against agricultural fungal pathogens.

Table 1 highlights not only the diversity of active constituents but also the complexity of their mechanism, which often involves multifactorial interactions rather than single-target effects. Phenolic compounds, such as eugenol, thymol, and carvacrol, predominantly disrupt fungal cell membranes and ion homeostasis, while aldehydes like citral and cinnamaldehyde interfere with ergosterol biosynthesis and mitochondrial respiration. Monoterpenes, such as linalool and geraniol, tend to impair cell wall synthesis and enzyme activity, and compounds like menthol and terpinen-4-ol increase membrane fluidity and oxidative stress. This diversity of biochemical targets is advantageous for agricultural use, as it minimizes the risk of fungal resistance and supports synergistic formulations combining different EO constituents.

Thymol, a phenolic compound classified as a monoterpene, present in thyme essential oil (*Thymus vulgaris*) and oregano (*Origanum vulgare*), interacts with fungal cell membranes, damaging the cellular structure, causing loss of ions, and leading to growth inhibition and cell death. A study by Martins et al. (2020)^40^ investigated the antifungal properties of oregano and thyme EOs against *Fusarium* species. The results indicated a high antifungal potential, with minimum inhibitory concentrations ranging from 0.078 to 0.313 μL/mL. Additionally, the combination of these oils exhibited synergistic interactions, enhancing environmental efficacy against pathogenic fungi.

Eugenol, a key component of clove EO, is widely studied for its fungicidal effects, including membrane permeabilization, inhibition of essential fungal metabolic enzymes, and disruption of mitochondrial respiration. A study by Milićević et al. (2022)^41^ examined the encapsulation of clove EO in various formulations and carriers, demonstrating high antifungal efficacy against *Botrytis cinerea*.

Citral, a monoterpenic aldehyde found in lemongrass (*Cymbopogon citratus*), is highly volatile and exhibits significant antifungal activity. Its primary mode of action involves interference with ergosterol synthesis, an essential component of fungal plasma membranes, leading to cellular disintegration and reduced pathogen viability. A study by Zhang et al. demonstrated citral’s potent antifungal effects against *Fusarium avenaceum*, where lemongrass EO at 0.3 μL/mL inhibited fungal growth through disruption of the plasma membrane and reducing pathogenicity by suppressing pectin methyl galacturonase activity.^42^

Carvacrol, a thymol isomer found in oregano and thyme EOs, disrupts the fungal lipid bilayer and impairs ion transport essential for cellular homeostasis. Its antioxidant activity also contributes to oxidative stress in pathogens, enhancing its antifungal action. Studies also suggest that carvacrol downregulates genes involved in ergosterol biosynthesis.^43,44^

Cinnamaldehyde, found in cinnamon EO *(Cinnamomum zeylanicum*), exhibits vigorous antifungal activity by inhibiting mitochondrial respiration and inducing oxidative stress in fungal cells. Wang et al. observed that cinnamaldehyde had a significant inhibitory effect on both mycelial growth and spore germination of *Fusarium solani*. Electron microscopy and propidium iodide staining revealed that cinnamaldehyde disrupted mycelial morphology, damaged plasma membranes, and interfered with ergosterol biosynthesis. Additionally, it increased ROS generation, leading to oxidative damage and compromising the integrity of pathogen cells. When applied in greenhouse conditions, cinnamaldehyde suppressed root rot in *Astragalus membranaceus* with 92% efficacy, highlighting its potential.^45^

Among EO constituents, the hydroxyl group (phenols) interacts with cell membranes, leading to the release of cellular components, modifying fatty acids and phospholipids, and interfering with energy metabolism, and influencing genetic material synthesis. The phenolic compounds such as eugenol, thymol, and carvacrol penetrate membranes, causing swelling, inhibiting respiratory enzymes, and dissipating the proton gradient and membrane potential.^46^

Overall, the antifungal mechanism summarized in Table 1 demonstrates that EO components act through complementary biochemical pathways, providing a rational basis for developing synergistic combinations or nanoencapsulated formulations that enhance stability and bioefficacy in agricultural environments. Despite their promising applications in agriculture, EOs have limitations due to their physical-chemical characteristics, including hydrophobicity, volatility, degradation, and sensitivity to light, heat, and oxygen.^47^ Carrier systems, including nanocapsules, nanoemulsions, and nanoparticles, have emerged as efficient strategies to optimize EO delivery, enhance stability, and minimize environmental impacts.^48^ Nanoencapsulation has been studied to protect EOs from premature degradation, thereby prolonging their biological activity through sustained and/or controlled release. Additionally, understanding the mechanism of action of key EOs components and the development of innovative delivery systems can significantly contribute to safer and more sustainable agricultural practices, reducing environmental impact and health risks associated with synthetic chemicals^49^

## 4 Nanotechnology for Enhancing Essential Oil Stability and Efficacy

EOs play essential roles in plant–arthropod interactions, including attracting pollinators and mediating the trophic activity of insect pests and their natural enemies. Additionally, they exhibit antimicrobial and insecticidal properties.^50–52^ Given these attributes, EOs have gained attention as environmentally friendly alternatives to conventional pest management strategies, reducing their ecological footprint.^53–55^

Despite their advantages, the practical application of EOs is hindered by intrinsic physicochemical limitations.^56^ EOs may exhibit phytotoxicity at high concentrations, and their efficacy in field applications is compromised by rapid degradation. Furthermore, their poor water solubility complicates the preparation of homogeneous aqueous solutions, further limiting their stability.^57^ However, nanotechnology can effectively address these challenges, which have emerged as a promising tool for developing bioactive formulations that enhance EO stability, sustained release, and efficacy.^58,59^

Among the different types of lipid-based formulations, nanoemulsions (NEs), nanostructured lipid carriers (NLCs), and solid lipid nanoparticles (SLNs) represent the main systems used for nanoencapsulation. As illustrated in Figure 2, these systems primarily differ in their internal structure and the ratio of liquid to solid lipids. Nanoemulsions are composed exclusively of liquid lipids, whereas SLNs are formed from solid lipids, resulting in a more crystalline matrix. In contrast, NLCs combine both solid and liquid lipids, forming a less-ordered core structure that allows higher loading of bioactive compounds and minimizes premature expulsion, while maintaining good colloidal stability and performance.

**Figure 2:**
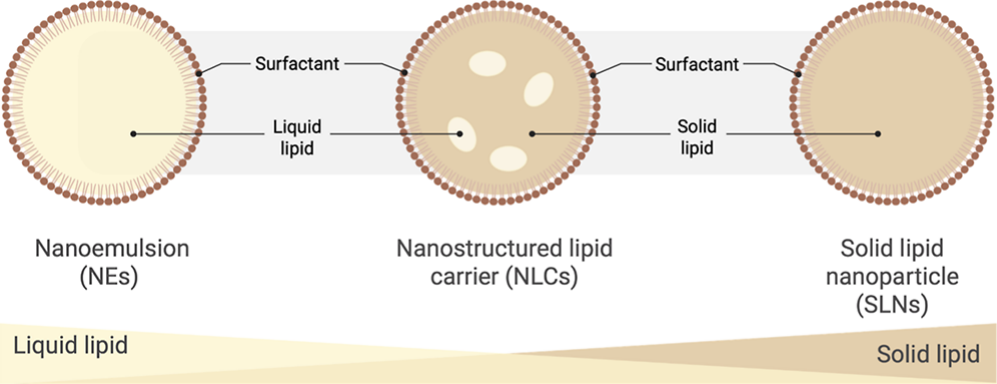
Schematic illustration of lipid-based nanocarriers: nanoemulsions (NEs), nanostructured lipid carriers (NLCs), and solid lipid nanoparticles (SLNs). The diagram highlights the fundamental structural differences in their lipid cores: NEs have a liquid lipid core, NLCs have a solid lipid matrix interspersed with liquid lipid pockets, and SLNs have an entirely solid lipid core. All three systems are stabilized by an outer layer of surfactant molecules. The gradient bar at the bottom conceptually illustrates the transition from a purely liquid lipid core (NEs) to a purely solid lipid core (SLNs), with NLCs representing an intermediate and more structured system. Created in Biorender.

The potential of lipid nanoformulations for EO encapsulation has earned considerable interest, particularly in the agri-food sector, due to their ability to stabilize volatile compounds, modulate release kinetics, and reduce the toxic effects on plants.^61^ Numerous studies have highlighted the effectiveness of EO-loaded lipid formulations in pharmaceuticals, cosmetics, and the food sector.^62–64^ Their application in crop preservation is especially promising (Table 2), as it could help reduce pesticide use and environmental contamination. However, additional research is needed to optimize lipid formulations for large-scale agricultural applications.^65–67^ Specifically, the transition from laboratory-scale proof-of-concept studies to commercially viable and environmentally robust formulations for widespread agricultural adoption presents significant hurdles, including scalability, cost-effectiveness, long-term shelf life, and sustained efficacy under variable field conditions.

**Table 2:** Overview of Studies on Essential Oil-Loaded Lipid-Based Nanocarriers to Control Fungi in Agriculture^a^ ^,^ ^b^

carrier system	essential oil	composition	physicochemical parameters (size, PDI, ζ-potential)	fungal target	crops	dose	potency parameter	Efectiveness	Study type	technology Readiness Level (TRL)	ref
SLN	Zataria multiflora EO	Thymol, Carvacrol, *p*-cymene	255.5 nm, 0.369, –37.8 mV	Alternaria solani, Rhizoctonia solani, Rhizopus stolonifer, *Aspergillus* spp	Tomato (Solanum lycopersicum)	100–1000 ppm	MIC = 200–50 ppm	High fungal inhibition, greater stability	in vitro	TRL 4	^68^
NLC	Cinnamomum zeylanicum EO	Cinnamaldehyde	94 nm PDI and ζ not reported	Penicillium citrinum *and*P. expansum	Citrus reticulata	0.3 and 0.6 mg/mL	MIC = 1.0 mg/mL (NLC) vs 0.425 mg/mL (free EO); MFC = 1.5 mg/mL (NLC) vs 0.675 mg/mL (free EO)	Infection reduced, lower weight loss and no adverse effect on flavor; maintained color and texture	in vitro, in vivo	TRL 5	^69^
NE	Clove, Black seed, (Syzygium aromaticum, Nigella sativa) OE	Eugenol, Thymoquinon, p-cymene, Carvacrol	82.6–131.9 nm, PDI and ζ not reported	Botrytis cinerea	Cucumber (Cucumis sativus)	5000 ppm (1%)	*	Complete suppression of seed rot and mortality, gray mold reduction on fruits (postspray) comparable to fungicide with no phytotoxicity	In vitro; In vivo (foliar application under natural infestation)	TRL 4	^70^
SLN	Oreganum vulgare and Thymus vulgaris EO	Thymol, (γ-terpinene),γ-terpinene, Endoborneol, *o*-cymene	180–188 nm 0.21–33 mV	Botrytis cinerea, Penicillium expansum	Not crop-specific	1000 μL L (0.1%)	*	Reduced mycelial growth, spore germination and more stable release	in vitro;	TRL 4	^71^
NE	Nigella sativa EO	-	168–350 nm 0.181–0.350 –48.8 mV	Penicillium verrucosum	Maize (Zea mays L.)	25–100%	*	Complete inhibition of P. verrucosum at 100%; improve seed germination	in vitro; in vivo	TRL 4	^72^
NE	Cymbopogon citratus, EO	Neral, Geraniale, Estragole, Geraniol	74.2 nm 0.19 –38.4	Penicillium digitatum, P. expansum	Citrus sinensis	4%–0.01% v/v	MIC = 0.03% (P. digitatum), 0.12% (P. expansum); MFC = 0.03–0.12%	Complete inhibition of A. niger and P. digitatum; strong reduction of fungal spread on coated fruits	in vitro, in vivo	TRL 5	^73^
NE	Citrus limon EO	Neral, Geranial, Linalool	103 nm	Penicilium digitatum, P. expansum	Citrus sinensis	4%–0.01% v/v	MIC = 0.03% (P. digitatum), 0.12% (P. expansum); MFC = 0.03–0.12%	Significant reduction of decay and weight loss; no sensory alteration	in vitro, in vivo	TRL 5	^73^
NLC	Lippia origanoides EO	Glyceryl distearate, Oleic acid, Thymol	156–190 nm, 0.2, 30| mV	Malassezia furfur, Aspergillus flavus, Fusarium keratoplasticum, Trichophyton rubrum	Not crop-specific	1–2%	MIC range = 24–1500 μg mL^–1^; thymol equivalent 3.9–142.6 μg mL^–1^	Strong antifungal activity	in vitro	TRL 4	^74^
Microemulsion (BLEO-ME)	Betel (*Piper betle* L.) EO	Chavibetol, Estragole, β-Cubebene, Chavicol, Caryophyllene	23.96 nm (oil:surfactant = 1:3 v/v, 5% oil); 0.482	Aspergillus flavus (MTCC 6750)	Tomato (Solanum lycopersicum)	0.2–3.0 ppm (corrected to mg/g per corrigendum)	*E*_max_ = 2.01 mg/g; MIC = 3.55 mg/g	Complete inhibition of A. flavus at ≥2.0 mg/g; extended lag time and reduced growth rate; higher activity	in vitro; in situ	TRL 4	^75^
NE	Satureja hortensis L. EO	Carvacrol, Thymol	41.72 nm, 0.291, –39.4 mV	Aspergillus parasiticus and Penicillium verrucosum	Not crop-specific	1%	MFC = 0.71 ± 0.05 mg L^–1^ (MF-ASHEO) vs 1.21 ± 0.15 mg L^–1^ (ASHEO)	Inhibited the growth of the fungal target	In vitro	TRL 4	^76^
NE	Syzygium aromaticum EO	Eugenol, Phenylpropanoid	80 – 110 nm	Fusarium oxysporum *f*. *sp*. *vasinfectum*	Cotton (*Gossypium* spp.)	1%, 2%, and 5% (v/v)	*	Higher inhibition of *Fusarium* mycelial growth	in vitro	TRL 5	^77^
NE	Lemongrass (Cymbopogon schoenanthus) EO	Citral, α-citral, β-citral, *d* -limonene, Geraniol	19.2 nm, 0.63, –34 mV	Aspergillus flavus	Not crop-specific	0.03–0.1% (v/v)	MFC ≈ 1 mg/mL (vs pure EO = 2 mg/mL); MGI up to 100%	Higher antifungal activity, inhibiting mycelial growth and reducing colony pigmentation	in vitro	TRL 4	^78^
NE	Thymus vulgaris	Thymol	61.45, 0.53, ζ not reported)	Botytis cinérea	Strawberry (*Fragaria* × ananassa)	0.5% (v/v)	MIC = 0.021%; MFC = 0.021%	Reduced decay, preserved firmness, increased vitamin C content, antioxidant activity	in vitro, in vivo	TRL 5	^79^
NE	Menta longifolia	4-methyl-2-(3-methyl-2-butenyl)-furan, Piperitenone oxide	60.51 0.51 ζ not reported)	Botytis cinerea	Strawberry (*Fragaria* × *ananassa*)	0.5% (v/v)	MIC = 0.021%; MFC = 0.021%	Reduced deterioration; lower microbial load; firmness	in vitro, in vivo	TRL 5}	

a Dose values represent the concentrations of essential oil–based formulations tested against the target fungi, expressed in parts per million (ppm), percentage (% v/v or w/v), or mass-to-volume ratios (μg mL^–1^ or mg mL^–1^), as reported by the authors. Potency parameters were expressed as the Minimum Inhibitory Concentration (MIC), i.e., the lowest concentration required to inhibit visible fungal growth, and/or the Minimum Fungicidal Concentration (MFC), i.e., the lowest concentration able to kill the fungus. When both parameters were determined, an MFC/MIC ratio ≤4 indicates antifungal activity. Additional parameters include *E*_max_ (maximum inhibitory effect) and MGI (mycelial growth inhibition). All values are reported as originally presented in each study. An asterisk (*) indicates information not reported in the source article.

b This table summarizes relevant research on different types of nanocarriers that encapsulate essential oils and their intended applications.

For example, Tortorici et al. developed a bioinsecticide formulation using NLCs loaded with EOs from rosemary, lavender, and peppermint. NLCs were prepared using Softisan 100 as the solid lipid, combined with a surfactant mixture of Kolliphor RH40 and Labrafil, both chosen for their environmentally friendly properties. The study evaluated the efficacy of the formulations against pests with different feeding strategies: *Aphis gossypii* (a sap-sucking aphid), *Spodoptera littoralis* (a leaf-chewing caterpillar), and *Tuta absoluta* (a leaf-mining moth). All formulations have a nanoparticle size of approximately 200 nm, a polydispersity index of less than 0.3, and exhibit colloidal stability for over 30 days. Bioassay results demonstrated that all EO-loaded formulations caused high mortality in *A. gossypii* and significantly reduced its reproductive capacity upon topical application. For *S. littoralis*, lavender- and rosemary-loaded NCLs reduced feeding without affecting survival. In contrast, none of the EO-loaded NCLs affected the survival or feeding behavior of *T. absoluta*.^79,80^ While demonstrating promising in vitro efficacy against specific pests and stable colloidal properties, inconsistent activity across different feeding strategies highlights a challenge for broad-spectrum application. Furthermore, the 30 day stability, while acceptable for some applications, may be insufficient for the extended shelf life often required in agricultural supply chains. The environmental friendliness of surfactants, while noted, also warrants further economic and scalable evaluation for large-scale production, especially considering the variability in raw material costs and availability.

Similarly, Hosseinpour Jajarm et al. developed SLNs using glyceryl palmitostearate (5% w/v) as the lipid phase, loaded with *Ziziphora clinopodioides* EO using combined homogenization and ultrasound techniques. The optimized formulation (2.5% EO) had a particle size of 241 nm, a zeta potential of −22.6 mV, and an encapsulation efficiency of 93%. Fumigant toxicity tests against *Tribolium castaneum* showed that EO-loaded SLN (LC_50_ 30.6 μL/L air) was more effective than pure oil (LC_50_ 68.3 μL/L air) and remained active for 14 days, while the pure oil lost toxicity after 8 days. Chemical analysis identified pulegone (51.78%) as the primary component.^81^ This study effectively demonstrates the potential for enhanced, prolonged pest control through nanoencapsulation, extending activity from 8 to 14 days. However, the application against a stored-product pest (*T. castaneum*) in fumigant tests limits direct extrapolation to open-field agricultural scenarios, where factors such as rapid volatilization, UV degradation, and rainfastness are more critical. Additionally, the combined homogenization and ultrasound technique, while effective for laboratory scale, presents notable challenges regarding energy consumption and batch consistency for industrial-scale production.

Polymeric coatings have been explored as an additional protective strategy to further improve the stability and functionality of EO-loaded NLCs. For example, Bashiri et al. successfully developed chitosan-coated NLCs via hot homogenization for the encapsulation of cinnamon EO. These coated nanoparticles demonstrated enhanced resistance to aggregation and oxidative degradation, maintaining an encapsulation efficiency above 84% even after 30 days of storage. The presence of chitosan provided a positive surface charge, stabilizing the system and facilitating interactions with negatively charged EO components, thereby enhancing oxidant activity for up to 21 days.^82^ While the use of chitosan coating offers improved colloidal stability and antioxidant activity, the economic viability and scalability of hot homogenization, coupled with the potential cost of high-purity chitosan for large-scale agricultural applications, are significant practical considerations. Moreover, the enhanced antioxidant activity, while beneficial for preserving EOs, does not directly translate into sustained antifungal efficacy and would require separate, rigorous evaluation in relevant agricultural models.

Plant-derived lipids include a variety of compounds, such as fatty acids, waxes, and isoprenoids.^83^ The composition and properties of these lipids can vary significantly and are influenced by genetic traits and environmental factors. Due to their natural origin, plant-based lipids present distinct advantages as they are biocompatible, nontoxic, and biodegradable, making them a safer alternative to synthetic lipids.^84^ Additionally, these lipids offer multiple benefits, including their ability to be functionalized for targeted delivery, their protective role in shielding bioactive compounds from environmental degradation, and the potential to increase sustainable production. Compared to lipids derived from animal sources, plant-based lipids enable the development of more eco-friendly lipid nanoparticles.^85^ Furthermore, their unique composition enhances NLC performance by providing lipid matrices with lower melting points, thereby enhancing drug loading capacity and modulating the release profile of encapsulated compounds. However, the inherent variability in the composition and properties of plant-derived lipids, driven by genetic and environmental factors, poses a significant challenge to achieving batch-to-batch consistency and reproducibility in large-scale industrial production. This variability necessitates stringent quality control measures and can complicate regulatory approval for agricultural applications. While promising for sustainable production, the actual scalability and consistent supply of specific high-quality plant lipids also require more comprehensive assessment.

For instance, Keivani Nahr et al. developed NLCs loading cardamom EO using cocoa butter as a solid lipid and olive oil as a liquid lipid, stabilized with Tween 80. The formulation was synthesized using low-energy emulsification, combined with high-shear homogenization and sonication. The nanoparticles size was below 150 nm, and the encapsulation efficiency was above 90%. Despite demonstrating improved stability of antimicrobial activity against model bacterial pathogens over 30 days, the relevance of these specific test organisms (*E. coli* and *S. aureus*) to agricultural fungal disease control is limited, highlighting a gap in directly applicable antifungal data. The synthesis method involving high-shear homogenization and sonication, while yielding well-characterized nanoparticles, also presents scalability and energy intensity challenges for agricultural applications.^86^

In another study, Pirouzifard et al. focused on encapsulating *Cuminum cyminum* EO in cocoa butter and cocoa butter substitute to improve its solubility, bioavailability, and stability. The NLCs were prepared via sonication-assisted homogenization, yielding particles <150 nm and an encapsulation efficiency >80%. The formulation showed good stability over one month, with minimal changes in particle size and the physical properties of the essential oil.^158^ While this study demonstrated formulation stability and effective encapsulation of hydrophobic EO, it did not address scalability or cost-efficiency, which are key barriers for agricultural translations. The use of food-grade lipids, such as cocoa butter, is advantageous for safety, but their cost and melting points may not be optimal for outdoor conditions.

Sivalingam et al. developed a biofungicide using NLCs loaded with clove essential oil, prepared via a hot-melt ultrasonication technique, formulated with glycerol monostearate as the solid lipid and coconut oil as the liquid component, and stabilized with lecithin and Tween 80. The optimized formulation exhibited an average size of 158 nm and a narrow size distribution (0.36), thereby enhancing the antifungal activity against *Fusarium oxysporum* compared to nonencapsulated EO. When comparing 250 ppm of EO-loaded NLCs with pure EO, the nanoformulation significantly inhibited the mycelial growth (88%) compared to the nonencapsulated EO (22%). The enhanced antifungal activity may be attributed to the small particle size and the increased surface-to-volume ratio, which likely improves their interaction with the fungal cell wall’s phospholipid bilayer and facilitates deeper penetration into the cytoplasm.^87^ This study provides compelling evidence for enhanced in vitro antifungal activity of encapsulated clove EO against a relevant agricultural pathogen (*F. oxysporum*). However, the “hot melt ultrasonication” technique can be energy-intensive and may pose scalability challenges, impacting the economic feasibility of large-scale production. Crucially, as an in vitro study focusing on mycelial growth inhibition, its findings, while promising, necessitate rigorous validation through in vivo and comprehensive field trials to account for environmental variables and complex plant–pathogen interactions.

More recently, Fuentes et al. produced SLNs using glyceryl tristearate as the lipid matrix via high-shear homogenization followed by ultrasonication, with *Mentha piperita* EO loaded. Nanoparticles were obtained with an average size of 200 nm and exhibited high thermal stability at 50 °C, resulting in a 70% reduction in *B. cinerea* growth, compared to 42% inhibition by blank SLNs. The EO altered fungal physiology by decreasing dry weight, increasing pH and electrical conductivity, and inducing oxidative stress.^88^ Similar to other examples, this study effectively demonstrates enhanced in vitro antifungal efficacy against *B. cinerea* and good thermal stability. Nevertheless, reliance on high-shear homogenization and ultrasonication again flags potential scalability and energy-cost issues. While thermal stability at 50 °C is beneficial, the performance and stability under other critical agricultural stresses, such as UV radiation, fluctuating humidity, and prolonged rain exposure, remain to be fully investigated. The in vitro nature of the efficacy assessment also leaves unanswered questions regarding the formulation’s performance in complex, dynamic field environments.

As shown earlier, the lipid-based formulations have emerged as a promising strategy for sustained delivery of EOs with antifungal activity in agricultural applications. These nanocarriers protect volatile bioactive compounds against environmental degradation while enabling more targeted and sustained release in the field.^61,89^ Although SLNs and NLCs are considered promising delivery systems, especially for the encapsulation of natural compounds, such as EOs, their application for fungal control in agriculture remains underexplored compared to their use in the biomedical sector. A critical comparison reveals that, despite compelling in vitro evidence for improved stability and efficacy, translating these laboratory successes into practical, scalable, and cost-effective agricultural solutions is largely hindered by persistent formulation challenges that receive insufficient attention in the current literature. There is also a significant dearth of studies utilizing EOs from diverse biodiversity, such as the rich Brazilian flora, which could offer novel and potent antifungal compounds.

In this context, we propose a modular and adaptable nanoplatform in which formulation parameters can be adjusted to suit the specific characteristics of the target fungus and/or crop. The idea of modulatory nanoplatforms relies on the ability to customize lipid composition, EO type and concentration, particle size, and surface modification, thereby creating a dynamic system that can be tailored to meet various agricultural needs. This approach is particularly interesting for controlling plant pathogenic fungi, which vary widely in their infection strategies, life cycle, sensitivity to natural compounds, and resistance mechanisms against conventional fungicides.^90–92^ Custom-designed nanoplatforms could be optimized for the control of phytopathogenic fungi, such as *Fusarium* spp., *B. cinerea*, *Puccinia* spp., *Colletotrichum* spp., and *Sclerotinia sclerotiorum*, which negatively impact the economy, while accounting for the physiological characteristics of different host crops. While theoretically appealing, the practical implementation of such a highly customizable modular nanoplatform faces considerable challenges, including the complexity of characterizing and validating an array of tailored formulations, the increased costs associated with specialized production runs, and the regulatory hurdles for approving diverse variants of a single “platform”. The current lack of systematic comparative studies on how different lipid matrices or EOs influence efficacy across varying agricultural environments further complicates this modular approach.

The versatility of the nanoplatform enables different formulations, for example, lipid-based formulations containing a single EO, which are suitable when the antifungal activity of a specific EO is well-established for the target fungi. On the other hand, combined EOs formulations with complementary mechanisms of action can broaden the antifungal spectrum and help delay the development of resistance.

Another promising approach involves the combination of EOs with biocontrol organisms, such as *Trichoderma* spp., *Bacillus* spp., *Pseudomonas fluorescens*, and *Beauveria bassiana*, resulting in a hybrid system that integrates chemical and biological modes of action.^91,93,94^ In such systems, encapsulation may protect sensitive microorganisms from EO toxicity, enabling their codelivery and combined action. However, the development of combined EO formulations, and particularly hybrid systems with biocontrol agents, introduces a new layer of complexity. Potential antagonistic interactions between EOs and beneficial microorganisms, precise control over release kinetics for each component, and the long-term stability of multicomponent systems are significant formulation challenges that are often overlooked in preliminary studies. Validating the synergistic effects and ensuring the viability of live biological agents within a nanocarrier system requires extensive research beyond current demonstrations.

Furthermore, the nanoplatform can target specific and/or different stages of fungal development. For example, fast-release formulations are better suited to inhibiting spore germination, a critical step in the early stages of infection for many phytopathogens. On the other hand, more sustained-release formulations are more effective in controlling hyphal growth over time, providing long-term protection throughout the crop growth. In advanced stages of infection, some fungi can form complex survival structures, such as biofilms, chlamydospores, or sclerotia, which are more challenging to eliminate. Functionalizing the nanoplatforms with enzymes, such as chitinases, can significantly improve the efficacy^95,96^ in these cases. While the concept of tailoring release profiles and functionalizing nanoplatforms with enzymes is highly innovative, it introduces considerable challenges, including formulation complexity, the stability of encapsulated enzymes (which are often sensitive to environmental factors), and the economic viability of producing such sophisticated systems on an agricultural scale. Moreover, demonstrating the selective action and persistence of these functionalities in diverse field conditions requires substantial further investigation.

Although many studies have demonstrated that lipid-based formulations containing EOs show promising results for controlling various fungal pathogens, most of these studies remain limited to in vitro assays. These studies often evaluated antifungal activity under controlled laboratory conditions, which may not accurately represent the complexity of field environments. Therefore, caution is required when assuming that in vitro results will translate directly into in vivo or field performance, as several factors, such as temperature fluctuations, solar radiation, rainfall, and interactions with soil and plant microbiota, can significantly influence the formulation’s stability and efficacy. For instance, Nie et al. demonstrated that a nanoemulsion formulated with *Foeniculum vulgare* essential oil exhibited strong antifungal activity against *F. oxysporum*, the causative agent of root-rot disease in *Panax notoginseng*. In vitro assays showed that the nanoemulsions had a minimum inhibitory concentration (MIC) of 0.35 mg/mL, approximately 10 times lower than that of the traditional essential oil formulation (3.65 mg/mL). For in vivo evaluation, the authors applied nanoemulsions at 0.5 mg/mL, a concentration slightly higher than the in vitro MIC, to infected *P. notoginseng* roots. Under these conditions, the lesion area was significantly reduced from ≈61.19 mm^2^ in the control group to ≈10.92 mm^2^, confirming a protective effect in planta.^97^ While this example effectively demonstrates a successful transition from in vitro to in planta efficacy, it is critical to highlight that even controlled in vivo experiments often fall short of replicating the full spectrum of environmental and biological variables encountered in open-field agriculture. Factors such as varying soil types, microbial communities, wind dispersion, and prolonged exposure to dynamic weather conditions can profoundly alter the performance and environmental fate of these nanoformulations. This crucial gap between laboratory and real-world field data remains a significant hurdle for widespread adoption and warrants more extensive, long-term, and multisite field trials.

While lipid-based nanoformulations of EOs have demonstrated promising antifungal activity both in vitro and in vivo, translating these results to field conditions may require careful optimization. Factors such as environmental variability, plant physiology, and potential volatilization or degradation of the active compounds can influence efficacy. Therefore, adjustments in concentration, formulation stability, and application methods are likely necessary to achieve the desired level of pest control under real agricultural conditions. Additionally, regulatory frameworks and cost-benefit analyses are rarely addressed, even though they are decisive factors in farmers technology adoption. Future work should thus integrate agronomic, economic, and environmental assessments to guide formulation development beyond the laboratory stage. Such optimization will be essential for the successful implementation of these sustainable antifungal strategies in the field. By leveraging Brazil’s rich biodiversity and biopolymer resources, future EO-loaded lipid nanoplatforms could uniquely combine ecological safety, local value generation, and high efficacy, contributing to a new generation of regionally tailored, climate-resilient agricultural technologies.

## 5 Application Strategies in Agriculture

Several practices are used in agricultural management to enhance crop growth, development, maintenance, and protection in the field, thereby increasing productivity (Figure 3). Seed treatment stands out as a key preplanting step, whether through industrial treatment or “on-farm” management.^98,99^ Foliar and soil applications are also frequently used throughout the crop cycle, mainly to control pests and diseases.^100^ Furthermore, in recent years, there has been growing concern about postharvest management (such as packaging development, processing steps, storage, and transportation), aiming to reduce losses, increase shelf life, product quality, and add value.^101^

**Figure 3:**
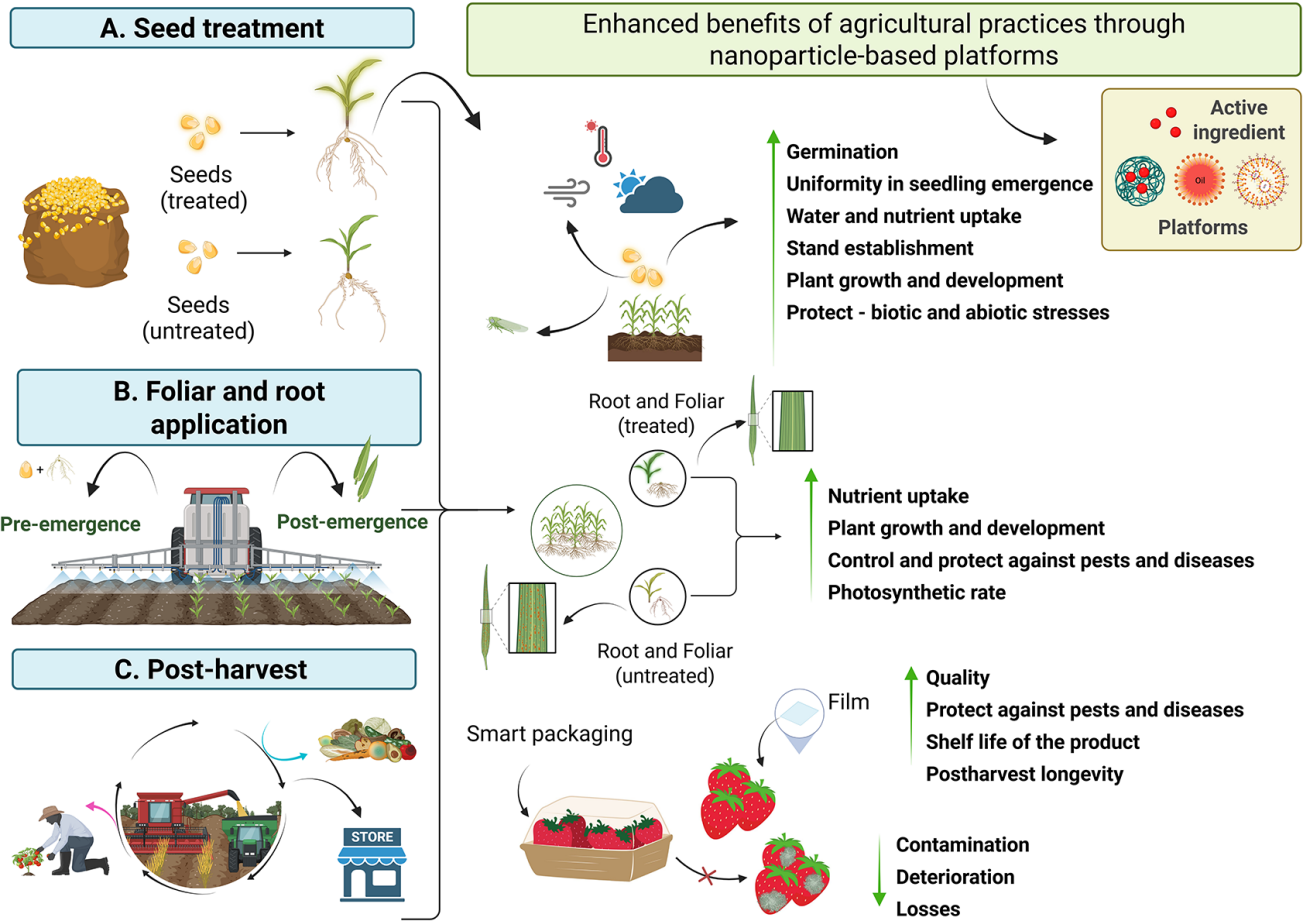
Nanoparticle-based platforms enhance agricultural practices across different crop production stages. The image illustrates the application of these platforms for seed treatment (A), foliar and root applications (B), and postharvest applications (C). At each phase, nanoenabled delivery of active ingredients contributes to improved plant growth, stress tolerance, pest and disease control, increased yield, and extended shelf life of harvested products. This approach supports more efficient and sustainable agricultural practices throughout the production chain.

Following the strategies depicted in Figure 3, nanotechnology emerges as a crucial ally in enhancing the efficiency and sustainability of agricultural management practices. It allows for the gradual and targeted release of formulations, protection against degradation, improved efficiency of compounds, and reduced environmental impacts.^102,103^

Seed treatment is a routine practice in integrated plant management. It consists of applying wet and/or dry chemical formulations (such as fungicides, insecticides, nematicides, macro and micronutrients, biostimulants) and biologicals (inoculants) around the seed, in the form of coating, film, pelleting or aggregation, acting as a tool in protecting plants against attacks by pests, diseases (in the field and during storage) and other adverse factors (excessive rain, drought, high and low temperatures, salinity, etc.) that may make the first stages of crop development in the field unfeasible. Additionally, this practice often results in increased germination, uniformity in seedling emergence, water and nutrient uptake, improved stand establishment, and plant growth and development.^104–106^

Guedes et al. reported that the emulsion with thyme essential oil inhibited spore germination and mycelial growth (EC_50_ = 0.05 mg/mL) of *F. oxysporum*, demonstrating its potential as a nanofungicide. Furthermore, seed treatment with the nanoemulsion did not negatively affect the physiological quality of the tomato seeds; on the contrary, it resulted in high germination and viability rates.^107^

The EO-loaded NLC formulations exhibited high thermal stability, low toxicity in *Galleria mellonella* larvae, and strong in vitro antifungal activity. The inclusion of the cationic surfactant (CTAB) significantly enhanced efficacy against most fungal species, due to a synergistic effect with thymol and to modifications in nanoparticle surface charge. However, *Candida auris*, *Aspergillus flavus*, and *Fusarium keratoplasticum* were not sensitive to CTAB, indicating that surface charge was not a determining factor for these species. Overall, the EO-loaded NLCs demonstrated promising potential as alternative antifungal agents.^74^

Foliar application is a common practice in crop management to meet the plant’s nutritional requirements, control and protect against pests and diseases, and mitigate the effects caused by biotic and abiotic stresses. The application involves the homogeneous spraying of the aerial part of plant species and has been consolidated as an efficient practice for the application of nanoparticles.^108,109^ Hong et al. describe that foliar application of nanoparticles can increase the effectiveness of plant protection technologies compared to soil/root application, but that it is necessary to understand in more detail essential factors that limit the interaction, such as morphophysiological characteristics of leaves, such as the presence of trichomes, hydathodes, cuticle thickening and cuticle composition, and metabolism of plant species.^109^ Furthermore, it is worth noting that interactions between the different nanomaterials and the functional groups on the leaf surface of each species (via hydrogen or covalent bonds, electrostatic or hydrophobic interactions) directly influence adhesion, absorption, distribution, and transport.^110^

Lipid nanoemulsions loaded with essential oils of pepper, garlic, and cinnamon positively affected antifungal activity against *Alternaria alternata* without presenting deleterious effects (morphological and physiological) to the culture.^111^ Studies with chitosan nanoparticles containing zinc applied to seeds and leaves in corn-controlled leaf spot disease (*Curvularia*), in addition to increasing grain yield. In this study, the application of nanoemulsions showed a 1.2–2.0-fold increase in superoxide dismutase (SOD) activity. Infected leaves exhibited white, circular spots with brown marginal rings that evolved into larger lesions. The other treated showed almost no symptoms of the disease, with only small lesions and no visible leaf necrosis, validating the effectiveness of both management practices.^112^ This provides essential insights into viable platforms for the application of biofungicides in the control of late blight and against economically essential pathogens in agriculture.^113^

Still aiming to make management more efficient, adopting technologies such as drones, sensors, and machinery with robotic systems can minimize daily challenges and waste, maximize efficiency, and reduce damage linked to soil compaction, for example, in both conventional systems and organic agriculture. In foliar applications, the adoption of drones has been increasing precisely because they enable the coupling of precision spraying systems capable of delivering pesticides, herbicides, or fertilizers with greater autonomy, uniformity, and precision. In addition, drones can be equipped with environmental sensors that provide information on crop microclimatic conditions, thereby optimizing management to more reliably prevent the spread of diseases. Therefore, they are an important technology for the management of nanoencapsule formulations.

In soil and root applications, soil characteristics (e.g., organic matter, clay, cation exchange capacity, pH, and microbiota) must be considered when applying nanomaterials. This interaction can result in aggregation, adsorption to soil colloids, and low absorption and transport of agrochemicals, thus altering their properties and functionalities.^114–118^ Zhao et al. demonstrated, for example, that the absorption of nanomaterials by the root is more limited in sandy soils due to aggregation with soil colloids.^119^

However, NPs and nanomaterials can generally be directed to the roots through the mass flow of water driven by transpiration or by diffusion along a concentration gradient. After being adsorbed to the superficial layer of the root (and by the roots), the particles can move along the cortex through the apoplastic or symplastic pathways, and be transported to the other parts of the plant.^120–122^

The use of nanoplatforms for the application of fungicides targeting soil pathogens can help maximize control and reduce losses in productivity, thereby reducing the risk of environmental damage by minimizing the amount of products available in the soil. Bueno et al. report that nanoencapsulation of azoxystrobin mitigates phytotoxic effects compared to the free molecule, allowing higher fresh mass content to be detected up to 20 days after exposure.^123^

At the postharvest and processing point, more than 40% of food (cereals, fruits, tubers etc.) is lost.^4^ This occurs due to poor quality during transportation, processing, storage, incorrect handling during harvesting, contamination, and deterioration caused by pests and pathogens, among numerous other factors. In this sense, nanotechnology can help mitigate these losses, whether through the application of nanoformulations, the development of innovative packaging, edible coatings, or the use of sensors. Edible nanocoatings, for example, in fruits, reduce dehydration levels and respiration rates, help maintain gas exchange and preserve volatile aromatic compounds, and reduce the risk of microbial development.

Thus, to meet production demands, food production requires the use of new technologies, such as nanotechnology, which can optimize current systems and minimize damage through more sustainable management.

## 6 Regulatory and Market Perspective

As demonstrated in this review, nanopesticides, particularly lipid-based nanoplatforms represent a significant advancement in sustainable agriculture by enhancing the antifungal activity and stability of essential oils while reducing environmental impacts.^80,124^ With a projected compound annual growth rate (CAGR) of 13.9% between 2024 and 2032, the global nanopesticide market, which stood at US$735 million in 2024, is expected to grow to around US$2.08 billion by 2032.^125,126^ This growth is pushed by increasing demand for eco-friendly agricultural solutions and regulatory restrictions on synthetic fungicides.^127^

Although many studies have demonstrated the efficacy of nanobased formulations loaded with essential oils for controlling fungal diseases in agriculture, their commercialization faces significant regulatory hurdles. Nanotechnology in agriculture, particularly for pest control and plant protection, is governed by evolving regulatory frameworks, which vary significantly worldwide. The regulatory approaches are primarily shaped by concerns regarding human health, environmental safety, and efficacy validation of nanobased formulations.^128^ For instance, in the United States, the Environmental Protection Agency (EPA) oversees the registration of nanopesticides under the Federal Insecticide, Fungicide, and Rodenticide Act (FIFRA), which requires extensive toxicity and environmental impact assessments. Similarly, the European Union (EU) has established regulations under the European Food Safety Authority (EFSA) for nanomaterials intended for food and agricultural applications, requiring detailed risk assessments and safety evaluations.

One of the main obstacles to the commercialization of views is the lack of standardized definitions of nanomaterials, which complicates regulatory compliance. In addition, the potential environmental and health risks associated with the accumulation of nanoparticles in soil and water ecosystems require long-term studies to assess their ecological impacts.^129^ Additionally, the high costs and complexity of the regulatory approval process deter small and medium-sized companies from investing in nanotechnology-based formulations for agricultural applications.^130^ Addressing these regulatory gaps through clear, standardized guidelines and risk-assessment protocols is crucial to simplifying the commercialization of nanobased formulations.

Consumer perception is crucial for the widespread adoption of nanobased formulations in agriculture. Essential oils are generally recognized as natural and eco-friendly alternatives to synthetic fungicides, driven by the growing interest in sustainable agricultural practices. On the other hand, the association with nanotechnology can raise concerns about potential toxicity and environmental risks.^131^ In this way, effective communication and labeling strategies that highlight the benefits of nanotechnology can overcome skepticism and build market confidence.^132^

Given this scenario, commercializing nanoplatforms based on lipids to deliver essential oil in agriculture requires standardized protocols for efficacy evaluation and testing their risk assessment. Nowadays, harmonized guidelines for evaluating the performance and safety of nanoformulations in agriculture^133,134^ are lacking. The efficacy of NLCs and SLNs in controlling fungal diseases should be tested under realistic field conditions, accounting for natural climate variability and crop-specific requirements. In addition, these protocols should address the potential toxicity of these nanoformulations, bioaccumulation, and adverse effects on nontarget organisms, such as beneficial organisms and soil microbiota.^135,136^

Standardized protocols would facilitate regulatory approval and allow comparative analysis of different nanoformulations, helping innovation and quality control.^137^ International bodies such as the International Organization for Standardization (ISO), the Organization for Economic Co-operation and Development (OECD), the European Food Safety Authority (EFSA), and the US Environmental Protection Agency (EPA) are actively developing and updating test guidelines and risk assessment frameworks for nanomaterials in agricultural applications.^137,138^ Despite these efforts, further harmonization is still required to address the unique properties of nanoscale formulations. Moreover, it is important to clearly distinguish between nanopesticides, which use a nanoscale delivery system for conventional actives; biopesticides, which are based on biological control agents or natural metabolites; and essential oils, which are generally recognized as safe (GRAS) but may still face regulatory scrutiny when nanoencapsulated.

The regulatory and market landscape for lipid nanoplatforms in agriculture presents both challenges and opportunities for essential oils. While regulatory uncertainties and consumer skepticism are significant barriers to widespread utilization, the growing demand for sustainable and effective fungicides underscores the potential for market growth. In summary, addressing these issues through a standardized regulatory framework, efficacy testing protocols, and public education will be crucial to unlocking the full potential of these innovative nanoformulations.

## 7 Future Directions and Challenges

It has been demonstrated that lipid-based nanoplatforms, such as nanostructured lipid carriers and solid lipid nanoparticles, for the delivery of essential oils to control fungal diseases in agriculture show promising results; however, many knowledge gaps and challenges remain that should be addressed to fully understand their potential.^139,140^ One of the biggest challenges is the large-scale production of these lipid nanoplatforms. While laboratory-scale production is well established, scaling up to industrial levels still faces technical barriers related to maintaining consistent physicochemical characteristics such as particle size and size distribution, encapsulation efficiency, and stability over time.^139–141^

In addition to the technical hurdles, the economic and operational feasibility of large-scale manufacturing remains a decisive limitation for its widespread adoption in agriculture.^142^ Conventional production methods, including high-pressure homogenization and ultrasonication, require expensive equipment, high-purity raw materials such as lipids and surfactants, and substantial energy input. These requirements result in elevated costs that are incompatible with the agricultural sector’s profit margins. Therefore, developing cost-effective, energy-efficient synthesis routes that maintain reproducibility and product quality is essential. Emerging alternatives, such as microfluidization, solvent-free synthesis, or continuous flow systems, may offer viable solutions for industrial scalability.Moreover, cost modeling studies indicate that lipid-based nanosystems often present production costs several-fold higher than conventional formulations, mainly due to purification steps, raw material purity requirements, and energy-intensive processes, reinforcing the need for low-cost lipids, scalable surfactants, and simplified downstream processing to enable commercial adoption.^143^

Another critical aspect concerns the physicochemical stability of these systems during storage, transportation, and field application. Temperature fluctuations, humidity, and light exposure can cause nanoparticle aggregation or premature release of essential oils, thereby reducing efficacy. Approaches such as lyophilization, the incorporation of natural stabilizers, or surface functionalization with biocompatible polymers, such as chitosan, pectin, cellulose, PEG derivatives and so on, can mitigate these effects and prolong shelf life. In parallel, large-scale drying methods, such as spray-drying and freezing drying, have been studied to improve stability, however, they may promote particle aggregation and size increase.^144,145^ To address this limitation, thermal protectants are commonly used to immobilize nanoplatforms with a glassy matrix. However, their stabilizing effect is concentration-dependent, and excessive amounts may affect the suspension stability of lipid-based nanoplatforms.^145^

Despite these efforts, long-term stability under real agricultural conditions remains insufficiently explored. Most studies have been conducted under controlled laboratory environments, while factors such as UV radiation, rainfall, and soil composition can significantly alter nanoplatform integrity and the kinetics of essential oil release.^146,147^ The use of polymeric coatings, such as polyethylene, poly­(vinyl alcohol), gelatin, or chitosan, has shown potential to enhance stability and compatibility,^148–151^ but additional research is needed to validate these strategies under open-field conditions. Furthermore, the environmental fate of these nanoplatforms and their interaction with nontarget organisms, including soil microbiota and beneficial insects, require further assessment to ensure ecological safety.^152–154^ Importantly, the regulatory landscape for nanoenabled agricultural inputs remains fragmented across regions, with agencies such as EFSA, EPA, and OECD calling for nanospecific risk-assessment frameworks but lacking standardized guidelines for registration. This regulatory uncertainty increases time-to-market and development costs and demands that future research address data gaps related to toxicokinetic, residue behavior, environmental persistence, and exposure assessment to comply with emerging nanosafety requirements.^155^

The application of lipid nanoplatforms containing EOs has demonstrated potential for controlling various fungal diseases, as evidenced by recent studies. However, it is crucial to recognize that, because of their broad spectrum of action, essential oils can negatively affect beneficial microorganisms in ecosystems. These microorganisms play vital roles, including improving nutrition through nitrogen fixation and nutrient release, providing protection against pathogens and pests and increasing tolerance to environmental stresses. Notably, to date, there is a distinct lack of published studies investigating the effect of essential oils nonencapsulated or when associated with lipid-based nanoplatforms on these beneficial organisms. Therefore, future research must be directed to a thorough evaluation of the negative impacts that these EOs and their derivatives, delivered via different lipid nanoplatforms, may exert on beneficial microbial populations, with the aim of minimizing undesirable side effects.

Another major gap lies in the validation of biological efficacy under field conditions. While many studies report excellent antifungal activity in vitro, data from greenhouse or open-field trials remain limited.^154,156^ For example, Fincheira et al. reported excellent reduction in spore germination of *B. cinerea* (80.9%) and *Penicillium expansum* (55.7%) treated with solid lipid nanoparticles loading essential oil of *T. vulgaris* at 15% v/v. Still, their efficacy in field conditions remains untested. In this way, future studies should focus on field trials to evaluate the antifungal activity and its impact on crop yield, plant growth, and soil health.^71^ Additionally, large-scale validation must consider variability in climate, crop management, soil type, and pest pressure, since several authors have shown that nanoformulations that perform well in laboratory or greenhouse settings often exhibit reduced or inconsistent activity in open fields due to environmental dilution, wash-off, or interactions with plant surfaces. As emphasized in recent reviews, multilocation, multiseason field trials remain a prerequisite for regulatory approval, risk assessment, and commercial deployment of nanoenabled agricultural technologies.^157^

To advance the practical and safe implementation of lipid-based nanoplatforms for EOs delivery in agriculture, future research should prioritize the following directions as showed in the Figure 4: (i) development of standardized release and stability testing protocols under conditions that mimic agricultural environments (e.g., UV exposure, temperature fluctuations, and soil interaction) to ensure consistent performance; (ii) establishment of target shelf life parameters and stability studies, similar to ICH guidelines, to facilitate product registration and commercial adoption; (iii) investigation of formulation compatibility and tank-mix behavior when lipid-based carriers are combined with commonly used agricultural inputs, avoiding degradation or antagonistic effects; (iv) Comprehensive evaluation of the potential effects on nontarget organisms and ecological balance, emphasizing interactions with beneficial microorganisms, pollinators, and soil fauna, as well as determining EOs toxicity threshold for microbial biocontrol agents such as Trichoderma; (v) assessment of application technology parameters, including droplet size, nozzle type, and application volume, to minimize spray drift, runoff, and losses during field application; (vi) definition of cost and scalability benchmarks, aiming to align nanoparticle production costs with acceptable thresholds for large-scale agriculture use; (vii) integration of nanospecific regulatory requirements into the development pipeline, including physicochemical characterization, nanospecific exposure assessment, and dossier preparation aligned with OECD test guidelines; (viii) expansion of field-scale validation protocols, incorporating multisite and multiseason designs to capture environmental variability and generate data sets suitable for regulatory evaluation and commercial scaling.

**Figure 4:**
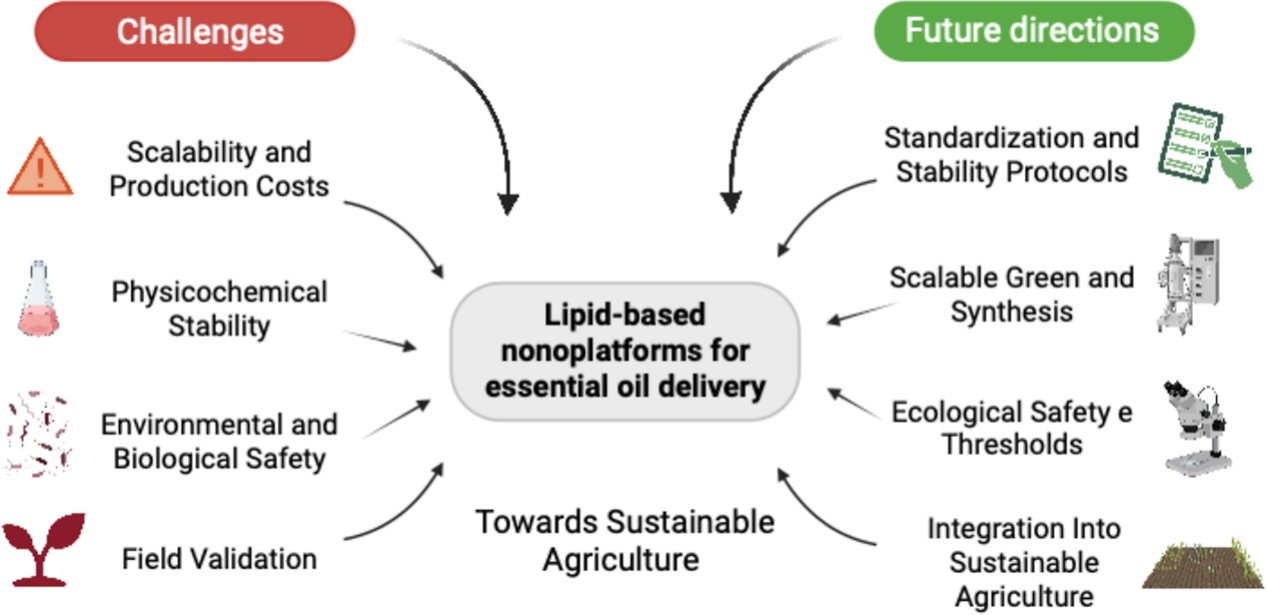
Schematic representation of the main challenges and future directions for the development of lipid-based nanoplatforms for essential oil delivery. Current limitations include scalability, production cost, physicochemical stability, and environmental and biological safety. Future perspectives highlight the need for standardized stability protocols, eco-friendly synthesis, ecological safety assessment, and field validation, with the aim of integration into sustainable agricultural systems.

Beyond these challenges, integrating lipid-based nanoplatforms with sustainable agricultural practices, such as biological control, crop rotation, and precision agriculture, represents a promising path forward. These nanoformulations enhance the stability and sustained release of bioactive compounds, reducing the need for synthetic pesticides and mitigating environmental contamination (SDG12: Responsible Consumption and Production). When combined with microbial biopesticides or biostimulants, they can also improve plant resilience and productivity, contributing to food security (SDG 2: Zero Hunger) and the preservation of soil and water quality (SDG 6: Clean Water and Sanitation).

In summary, lipid-based nanoplatforms for EOs delivery hold significant promise for sustainable fungal disease management. Yet, scalability, economic feasibility, long-term stability, regulatory uncertainties, and field-scale validation remain the main bottlenecks. By focusing on the above priorities, future research can accelerate the translation of laboratory success into reliable, field-ready technologies that are both economically and ecologically sustainable. Addressing these issues will require multidisciplinary collaboration across nanotechnology, agronomy, and environmental science to ensure that these systems become viable, safe, and effective tools for building more resilient, sustainable agriculture.

## 8 Conclusions

Lipid-based nanoplatforms offer a promising approach for the sustainable delivery of essential oils in agriculture, enhancing stability and reducing environmental impact. Despite the compelling scientific advancements, the current body of literature reveals a critical disparity: many studies prioritize proof-of-concept in vitro or small-scale in vivo demonstrations over comprehensive investigations into the practical, economic, and scalable aspects of formulation development for agriculture. A more rigorous, comparative analysis of formulation stability under harsh environmental conditions, alongside robust field trials, is indispensable for validating the long-term potential and viability of nanoplatforms to control antifungal diseases and promote a more sustainable agriculture.

## References

[1] Rezaei E. E., Webber H., Asseng S., Boote K., Durand J. L., Ewert F., Martre P., MacCarthy D. S. (2023). Climate
Change Impacts on Crop Yields. Nat. Rev. Earth
Environ..

[60] Nguyen H. M., Hwang I. C., Park J. W., Park H. J. (2012). Enhanced Payload
and Photo-Protection for Pesticides Using Nanostructured Lipid Carriers
with Corn Oil as Liquid Lipid. J. Microencapsulation.

[2] Zhou J., Zhang X., Qu Z., Zhang C., Wang F., Gao T., Yao Y., Liang J. (2024). Progress in Research on Prevention
and Control of Crop Fungal Diseases in the Context of Climate Change. Agriculture.

[3] Almeida F., Rodrigues M. L., Coelho C. (2019). The Still Underestimated Problem
of Fungal Diseases Worldwide. Front. Microbiol..

[4] Food and Agriculture Organization . Mudança Climática Influencia Na Perda Da Produção Agrícola Para Pragas, Conclui Estudo Apoiado Pela FAO, 2021. https://www.fao.org/brasil/noticias/detail-events/fr/c/1411810 (accessed 2025–08–05).

[5] Statista Research Department . Topic: Agricultural chemical industry worldwide; Statista. https://www.statista.com/topics/3062/agricultural-chemical-industry/ (accessed 2025–07–30).

[6] Companhia Nacional de Abastecimento . Boletim de Monitoramento Agrícola, 2025. https://www.gov.br/conab/pt-br/atuacao/informacoes-agropecuarias/safras/safra-de-graos/monitoramento-agricola/boletim-de-monitoramento-dos-cultivos-de-verao-abril-2025.pdf.

[7] Wu P.-H., Chang H.-X., Shen Y.-M. (2023). Effects
of Synthetic and Environmentally
Friendly Fungicides on Powdery Mildew Management and the Phyllosphere
Microbiome of Cucumber. PLoS One.

[8] Leoci R., Ruberti M. (2021). Pesticides: An Overview
of the Current Health Problems
of Their Use. J. Geosci. Environ. Protect..

[9] Ngegba P. M., Cui G., Khalid M. Z., Zhong G. (2022). Use of Botanical Pesticides in Agriculture
as an Alternative to Synthetic Pesticides. Agriculture.

[10] de
Sousa D. P., Damasceno R. O. S., Amorati R., Elshabrawy H. A., de Castro R. D., Bezerra D. P., Nunes V. R. V., Gomes R. C., Lima T. C. (2023). Essential Oils: Chemistry and Pharmacological Activities. Biomolecules.

[11] Barradas T. N., de Holanda e Silva K. G. (2021). Nanoemulsions
of Essential Oils to
Improve Solubility, Stability and Permeability: A Review. Environ. Chem. Lett..

[12] de
Oliveira J. L., Campos E. V. R., Bakshi M., Abhilash P. C., Fraceto L. F. (2014). Application of Nanotechnology for the Encapsulation
of Botanical Insecticides for Sustainable Agriculture: Prospects and
Promises. Biotechnol. Adv..

[13] Mall J., Naseem N., Haider M. F., Rahman M. A., Khan S., Siddiqui S. N. (2025). Nanostructured Lipid
Carriers as a Drug Delivery System:
A Comprehensive Review with Therapeutic Applications. Intell. Pharm..

[14] Ajala T. O., Abraham A., Keck C. M., Odeku O. A., Elufioye T. O., Olopade J. O. (2021). Shea Butter (*Vitellaria Paradoxa*)
and *Pentaclethra Macrophylla* Oil as Lipids in the
Formulation of Nanostructured Lipid Carriers. Sci. Afr..

[15] Meyer, M. C. ; Campos, H. D. ; Godoy, C. V. ; Utiamada, M. C. ; Sato, L. N. ; Dias, A. R. ; Junior, J. N. ; Junior, M. L. ; Tirmen, N. L. Eficiência de fungicidas para controle de mofo-branco (Sclerotinia sclerotiorum) em soja, na safra 2020/2021: Resultados Sumarizados dos Experimentos Cooperativos; Portal Embrapa. https://www.embrapa.br/busca-de-publicacoes/-/publicacao/1133378/eficiencia-de-fungicidas-para-controle-de-mofo-branco-sclerotinia-sclerotiorum-em-soja-na-safra-20202021-resultados-sumarizados-dos-experimentos-cooperativos (accessed 2025–07–30).

[16] Rathore S., Mukhia S., Kumar R., Kumar R. (2023). Essential Oil Composition
and Antimicrobial Potential of Aromatic Plants Grown in the Mid-Hill
Conditions of the Western Himalayas. Sci. Rep..

[17] Sadgrove N. J., Padilla-González G. F., Phumthum M. (2022). Fundamental Chemistry
of Essential Oils and Volatile Organic Compounds, Methods of Analysis
and Authentication. Plants.

[18] Rozwalka L. C., Moreira R. R., Ballesteros Garcia M.
J., Marques F. A., May De Mio L. L. (2020). Chemical Components of Essential Oils as a Base to
Control Two Grape Pathogens: Sphaceloma Ampelinum and Pseudocercopora
Vitis. J. Phytopathol..

[19] Jing C., Zhao J., Han X., Huang R., Cai D., Zhang C. (2018). Essential Oil of *Syringa Oblata* Lindl. as a Potential
Biocontrol Agent against Tobacco Brown Spot Caused by *Alternaria
Alternata*. Crop Prot..

[20] Nazzaro F., Fratianni F., Coppola R., Feo V. D. (2017). Essential
Oils and
Antifungal Activity. Pharmaceuticals.

[21] Yu D., Wang J., Shao X., Xu F., Wang H. (2015). Antifungal
Modes of Action of Tea Tree Oil and Its Two Characteristic Components
against *Botrytis Cinerea*. J.
Appl. Microbiol..

[22] Konuk H. B., Ergüden B. (2022). Investigation of Antifungal Activity Mechanisms of
Alpha-Pinene, Eugenol, and Limonene. J. Adv.
Vetbio Sci. Tech..

[23] Hilgers F., Habash S. S., Loeschcke A., Ackermann Y. S., Neumann S., Heck A., Klaus O., Hage-Hülsmann J., Grundler F. M. W., Jaeger K.-E., Schleker A. S. S., Drepper T. (2021). Heterologous
Production of β-Caryophyllene and Evaluation of Its Activity
against Plant Pathogenic Fungi. Microorganisms.

[24] Zhu Y., Li T.-T., Zhou S.-W., Qin X.-J., Li Y., Noiprasert S., Apiwongsrichai K., Xu F.-R., Liu X.-Y., Dong X. (2025). β-Caryophyllene
Regulates H3K36me3 to Inhibit Conidial Germination
and Mycelial Growth of Fusarium Proliferatum. BMC Microbiol..

[25] Rao A., Zhang Y., Muend S., Rao R. (2010). Mechanism of Antifungal
Activity of Terpenoid Phenols Resembles Calcium Stress and Inhibition
of the TOR Pathway. Antimicrob. Agents Chemother..

[26] Chavan P. S., Tupe S. G. (2014). Antifungal Activity
and Mechanism of Action of Carvacrol
and Thymol against Vineyard and Wine Spoilage Yeasts. Food Control.

[27] OuYang Q., Duan X., Li L., Tao N. (2019). Cinnamaldehyde Exerts
Its Antifungal Activity by Disrupting the Cell Wall Integrity of Geotrichum
Citri-Aurantii. Front. Microbiol..

[28] Shreaz S., Wani W. A., Behbehani J. M., Raja V., Irshad M., Karched M., Ali I., Siddiqi W. A., Hun L. T. (2016). Cinnamaldehyde
and Its Derivatives, a Novel Class of Antifungal Agents. Fitoterapia.

[29] Leite M. C. A., Bezerra A. P. D. B., Sousa J. P. D., Guerra F. Q. S., Lima E. D. O. (2014). Evaluation of
Antifungal Activity and Mechanism of
Action of Citral against *Candida Albicans*. Evid. base Compl. Alternative Med..

[30] Didehdar M., Chegini Z., Shariati A. (2022). Eugenol: A Novel Therapeutic Agent
for the Inhibition of Candida Species Infection. Front. Pharmacol.

[31] Vasconcelos P. G. S., Lee K. M., Abuna G. F., Costa E. M. M. B., Murata R. M. (2024). Monoterpene Antifungal Activities:
Evaluating Geraniol,
Citronellal, and Linalool on Candida Biofilm, Host Inflammatory Responses,
and Structure–Activity Relationships. Front. Pharmacol.

[32] Tang X., Shao Y.-L., Tang Y.-J., Zhou W.-W. (2018). Antifungal Activity
of Essential Oil Compounds (Geraniol and Citral) and Inhibitory Mechanisms
on Grain Pathogens (Aspergillus Flavus and Aspergillus Ochraceus). Molecules.

[33] Li X., Wang Q., Li H., Wang X., Zhang R., Yang X., Jiang Q., Shi Q. (2023). Revealing the Mechanisms
for Linalool Antifungal Activity against Fusarium Oxysporum and Its
Efficient Control of Fusarium Wilt in Tomato Plants. Int. J. Mol. Sci..

[34] Biernasiuk A., Malm A. (2023). Synergistic Interactions
between Linalool and Some Antimycotic Agents
against Candida Spp. as a Basis for Developing New Antifungal Preparations. Appl. Sci..

[35] Ben
Miri Y., Nouasri A., Herrera M., Djenane D., Ariño A. (2023). Antifungal
Activity of Menthol, Eugenol and Their Combination against Aspergillus
Ochraceus and Aspergillus Niger In Vitro and in Stored Cereals. Foods.

[36] Zore G., Thakre A., Abdulghani M., Bhosle K., Shelar A., Patil R., Kharat K., Karuppayil S. (2022). Menthol Inhibits
Candida Albicans Growth by Affecting the Membrane Integrity Followed
by Apoptosis. Evid. base Compl. Alternative
Med..

[37] Montenegro I., Said B., Godoy P., Besoain X., Parra C., Díaz K., Madrid A. (2020). Antifungal Activity of Essential
Oil and Main Components from Mentha Pulegium Growing Wild on the Chilean
Central Coast. Agronomy.

[38] Aimad A., Sanae R., Anas F., Abdelfattah E. M., Bourhia M., Mohammad Salamatullah A., Alzahrani A., Alyahya H. K., Albadr N. A., Abdelkrim A., El Barnossi A., Noureddine E. (2021). Chemical Characterization and Antioxidant,
Antimicrobial, and Insecticidal Properties of Essential Oil from Mentha
Pulegium L. Evid. base Compl. Alternative Med..

[39] De
Castro R. D., De Souza T. M. P. A., Bezerra L. M. D., Ferreira G. L. S., De Brito Costa E. M. M., Cavalcanti A. L. (2015). Antifungal
Activity and Mode of Action of Thymol and Its Synergism with Nystatin
against Candida Species Involved with Infections in the Oral Cavity:
An in Vitro Study. BMC Complementary Altern.
Med..

[40] Martins G. A., Bicas J. L. (2024). Antifungal Activity
of Essential Oils of Tea Tree,
Oregano, Thyme, and Cinnamon, and Their Components. Braz. J. Food Technol..

[41] Milićević Z., Krnjajić S., Stević M., Ćirković J., Jelušić A., Pucarević M., Popović T. (2022). Encapsulated Clove Bud Essential Oil: A New Perspective
as an Eco-Friendly Biopesticide. Agriculture.

[42] Zhang Z., Mo Z., Zhang X., Wang J., Li J., Shi H., Wang P., Lin Z. (2022). The Antifungal Activity and Action
Mechanism of Lemongrass (Cymbopogon Flexuosus) Essential Oil Against
Fusarium Avenaceum. J. Essent. Oil Bear. Plants.

[43] Ahmad A., Khan A., Akhtar F., Yousuf S., Xess I., Khan L. A., Manzoor N. (2011). Fungicidal
Activity of Thymol and
Carvacrol by Disrupting Ergosterol Biosynthesis and Membrane Integrity
against Candida. Eur. J. Clin. Microbiol. Infect.
Dis..

[44] Tópor
Nunes A. A., Veras F. F., Cacciatore F. A., Silveira R. D., Malheiros P. D. S., Welke J. E. (2025). Nanoencapsulation
with Eudragit® and Chia Mucilage Increases the Stability and
Antifungal Efficacy of Carvacrol against *Aspergillus* Spp. Food Addit. Contam.,: Part A.

[45] Wang J., Zhang J., Ma J., Liu L., Li J., Shen T., Tian Y. (2022). The Major Component
of Cinnamon Oil
as a Natural Substitute against Fusarium Solani on Astragalus Membranaceus. J. Appl. Microbiol..

[46] Di
Pasqua R., Betts G., Hoskins N., Edwards M., Ercolini D., Mauriello G. (2007). Membrane Toxicity of Antimicrobial
Compounds from Essential Oils. J. Agric. Food
Chem..

[47] Li Z., Jiang X., Liu H., Yao Z., Liu A., Ming L. (2022). Evaluation of Hydrophilic and Hydrophobic
Silica Particles on the
Release Kinetics of Essential Oil Pickering Emulsions. ACS Omega.

[48] Flores F. C., Ribeiro R. F., Ourique A. F., Rolim C. M. B., Silva C. d. B. d., Pohlmann A. R., Beck R. C. R., Guterres S. S. (2011). Nanostructured Systems
Containing an Essential Oil: Protection against Volatilization. Quím. Nova.

[49] Veloz V. A., Santos L. R. dos., Amaral J. G., Garcia L. B., Oliveira R. A., Santos R. L. S. R. (2024). Nanopartículas
de Zeína/Pva Incorporadas
Com Eugenol e Óleo Essencial do Cravo-Daíndia: OtimizaçÃo
da Síntese E ValidaçÃo Analítica Para
QuantificaçÃo do Eugenol. Quím.
Nova.

[50] Soares M. A., Campos M. R., Passos L. C., Carvalho G. A., Haro M. M., Lavoir A.-V., Biondi A., Zappalà L., Desneux N. (2019). Botanical Insecticide and Natural
Enemies: A Potential
Combination for Pest Management against Tuta Absoluta. J. Pest. Sci..

[51] Al-Ansari M. M., Andeejani A. M. I., Alnahmi E., AlMalki R. H., Masood A., Vijayaraghavan P., Rahman A. A., Choi K. C. (2021). Insecticidal
Antimicrobial
and Antioxidant Activities of Essential Oil from *Lavandula
Latifolia* L. and Its Deterrent Effects on *Euphoria
Leucographa*. Ind. Crops Prod..

[52] Benelli G., Ceccarelli C., Zeni V., Rizzo R., Lo Verde G., Sinacori M., Boukouvala M. C., Kavallieratos N. G., Ubaldi M., Tomassoni D., Benvenuti F., Roy P., Petrelli R., Cappellacci L., Spinozzi E., Maggi F., Canale A. (2022). Lethal and Behavioural
Effects of a Green Insecticide
against an Invasive Polyphagous Fruit Fly Pest and Its Safety to Mammals. Chemosphere.

[53] Andrade F. P., Venzon M., Das Dôres R. G.
R., Franzin M. L., Martins E. F., De Araújo G. J., Fonseca M. C. M. (2021). Toxicity of Varronia
Curassavica Jacq. Essential Oil to Two Arthropod Pests and Their Natural
Enemy. Neotrop. Entomol..

[54] Pavela R., Pavoni L., Bonacucina G., Cespi M., Cappellacci L., Petrelli R., Spinozzi E., Aguzzi C., Zeppa L., Ubaldi M., Desneux N., Canale A., Maggi F., Benelli G. (2021). Encapsulation of Carlina
Acaulis Essential Oil and
Carlina Oxide to Develop Long-Lasting Mosquito Larvicides: Microemulsions
versus Nanoemulsions. J. Pest. Sci..

[55] Sciortino M., Scurria A., Lino C., Pagliaro M., D’Agostino F., Tortorici S., Ricupero M., Biondi A., Zappalà L., Ciriminna R. (2021). Silica-Microencapsulated Orange Oil for Sustainable
Pest Control. Adv. Sustain. Syst..

[56] Tian Q., Zhou W., Cai Q., Ma G., Lian G. (2021). Concepts,
Processing, and Recent Developments in Encapsulating Essential Oils. Chin. J. Chem. Eng..

[57] Campolo O., Cherif A., Ricupero M., Siscaro G., Grissa-Lebdi K., Russo A., Cucci L. M., Di Pietro P., Satriano C., Desneux N., Biondi A., Zappalà L., Palmeri V. (2017). Citrus Peel Essential Oil Nanoformulations
to Control
the Tomato Borer, Tuta Absoluta: Chemical Properties and Biological
Activity. Sci. Rep..

[58] Achagar R., Ait-Touchente Z., El Ati R., Boujdi K., Thoume A., Abdou A., Touzani R. (2024). A Comprehensive Review
of Essential
Oil–Nanotechnology Synergy for Advanced Dermocosmetic Delivery. Cosmetics.

[59] Weisany W., Yousefi S., Tahir N. A., Golestanehzadeh N., McClements D. J., Adhikari B., Ghasemlou M. (2022). Targeted Delivery
and Controlled Released of Essential Oils Using Nanoencapsulation:
A Review. Adv. Colloid Interface Sci..

[61] Katopodi A., Detsi A. (2021). Solid Lipid Nanoparticles and Nanostructured
Lipid Carriers of Natural
Products as Promising Systems for Their Bioactivity Enhancement: The
Case of Essential Oils and Flavonoids. Colloids
Surf., A.

[62] Maroofpour N., Hejazi M. J., Hamishehkar H., Iranipour S. (2019). Relative Toxicity
and Residual Activity of Nanocapsules and Commercial Formulations
of Pirimicarb and Pymetrozine Against Myzus Persicae (Hemiptera: Aphididae). J. Econ. Entomol..

[63] Waghule T., Rapalli V. K., Gorantla S., Saha R. N., Dubey S. K., Puri A., Singhvi G. (2020). Nanostructured Lipid Carriers as
Potential Drug Delivery Systems for Skin Disorders. CPD.

[64] Dobreva M., Stefanov S., Andonova V. (2020). Natural Lipids as Structural Components
of Solid Lipid Nanoparticles and Nanostructured Lipid Carriers for
Topical Delivery. CPD.

[65] Frederiksen H. K., Kristensen H. G., Pedersen M. (2003). Solid Lipid Microparticle Formulations
of the Pyrethroid Gamma-Cyhalothrin—Incompatibility of the
Lipid and the Pyrethroid and Biological Properties of the Formulations. J. Controlled Release.

[66] Kumar A., Kanwar R., Mehta S. K. (2022). Recent Development
in Essential Oil-Based
Nanocarriers for Eco-Friendly and Sustainable Agri-Food Applications:
A Review. ACS Agric. Sci. Technol..

[67] Nguyen M.-H., Nguyen T.-H.-N., Hwang I.-C., Bui C.-B., Park H.-J. (2016). Effects
of the Physical State of Nanocarriers on Their Penetration into the
Root and Upward Transportation to the Stem of Soybean Plants Using
Confocal Laser Scanning Microscopy. Crop Prot..

[68] Nasseri M., Golmohammadzadeh S., Arouiee H., Jaafari M. R., Neamati H. (2016). Antifungal
Activity of Zataria Multiflora Essential Oil-Loaded Solid Lipid Nanoparticles
in-Vitro Condition. Iran J. Basic Med. Sci..

[69] Radi M., Ahmadi H., Amiri S. (2022). Effect of
Cinnamon Essential Oil-Loaded
Nanostructured Lipid Carriers (NLC) Against Penicillium Citrinum and
Penicillium Expansum Involved in Tangerine Decay. Food Bioprocess Technol..

[70] Ziedan E.-S. H. E., Saad M. M., El-Kafrawy A. A., Sahab A. F., Mossa A.-T. H. (2022). Evaluation
of Essential Oils Nanoemulsions Formulations on Botrytis Cinerea Growth,
Pathology and Grey Mould Incidence on Cucumber Fruits. Bull. Natl. Res. Cent..

[71] Fincheira P., Espinoza J., Levío-Raimán M., Vera J., Tortella G., Brito A. M. M., Seabra A. B., Diez M. C., Quiroz A., Rubilar O. (2024). Formulation of Essential
Oils-Loaded
Solid Lipid Nanoparticles-Based Chitosan/PVA Hydrogels to Control
the Growth of Botrytis Cinerea and Penicillium Expansum. Int. J. Biol. Macromol..

[72] Mosa M. A., Youssef K., Hamed S. F., Hashim A. F. (2023). Antifungal Activity
of Eco-Safe Nanoemulsions Based on Nigella Sativa Oil against Penicillium
Verrucosum Infecting Maize Seeds: Biochemical and Physiological Traits. Front. Microbiol..

[73] Gharzouli M., Aouf A., Mahmoud E., Ali H., Alsulami T., Badr A. N., Ban Z., Farouk A. (2024). Antifungal
Effect of
Algerian Essential Oil Nanoemulsions to Control Penicillium Digitatum
and Penicillium Expansum in Thomson Navel Oranges (Citrus Sinensis
L. Osbeck). Front. Plant Sci..

[74] Gil G. A., Kakuda L., Tonani L., Von Zeska
Kress M. R., Oliveira W. P. (2025). Surfactant-Driven Effects on the
Antifungal Activity
of *Lippia Origanoides* Kunth Essential Oil Encapsulated
in Lipid-Based Nanosystems. ACS Omega.

[75] Basak S., Guha P. (2017). Betel Leaf, (*Piper* Betle L.) Essential Oil Microemulsion:
Characterization and Antifungal Activity on Growth, and Apparent Lag
Time of Aspergillus Flavus in Tomato Paste. LWT.

[76] Boudechicha A., Aouf A., Ali H., Alsulami T., Badr A. N., Ban Z., Farouk A. (2024). Effect of
Microfluidization on the Volatiles and Antibacterial,
Antifungal, and Cytotoxic Activities of Algerian *Satureja
Hortensis* L. Oil-Loaded Nanoemulsions: *In Vitro* and *In Silico* Study. ACS
Omega.

[77] Abd-Elsalam K. A., Khokhlov A. R. (2015). Eugenol Oil Nanoemulsion: Antifungal Activity against
Fusarium Oxysporum f. Sp. Vasinfectum and Phytotoxicity on Cottonseeds. Appl. Nanosci..

[78] Liu L., Fisher K. D., Friest M. A., Gerard G. (2023). Characterization and
Antifungal Activity of Lemongrass Essential Oil-Loaded Nanoemulsion
Stabilized by Carboxylated Cellulose Nanofibrils and Surfactant. Polymers.

[79] Javanmardi Z., Koushesh Saba M., Nourbakhsh H., Amini J. (2023). Efficiency of Nanoemulsion
of Essential Oils to Control Botrytis Cinerea on Strawberry Surface
and Prolong Fruit Shelf Life. Int. J. Food Microbiol..

[80] Tortorici S., Cimino C., Ricupero M., Musumeci T., Biondi A., Siscaro G., Carbone C., Zappalà L. (2022). Nanostructured
Lipid Carriers of Essential Oils as Potential Tools for the Sustainable
Control of Insect Pests. Ind. Crops Prod..

[81] Hosseinpour
Jajarm F., Moravvej G., Modarres Awal M., Golmohammadzadeh S. (2021). Insecticidal Activity of Solid Lipid Nanoparticle Loaded
by *Ziziphora Clinopodioides* Lam. against *Tribolium Castaneum* (Herbst, 1797) (Coleoptera: Tenebrionidae). Int. J. Pest Manag..

[82] Bashiri S., Ghanbarzadeh B., Ayaseh A., Dehghannya J., Ehsani A. (2020). Preparation and Characterization of Chitosan-Coated
Nanostructured Lipid Carriers (CH-NLC) Containing Cinnamon Essential
Oil for Enriching Milk and Anti-Oxidant Activity. LWT.

[83] Jansen B., Wiesenberg G. L. B. (2017). Opportunities
and Limitations Related to the Application
of Plant-Derived Lipid Molecular Proxies in Soil Science. Soil.

[84] Sarvarian P., Samadi P., Gholipour E., Shams Asenjan K., Hojjat-Farsangi M., Motavalli R., Motavalli Khiavi F., Yousefi M. (2022). Application of Emerging Plant-Derived
Nanoparticles
as a Novel Approach for Nano-Drug Delivery Systems. Immunol. Investig..

[85] Tinnirello V., Rabienezhad Ganji N., De Marcos Lousa C., Alessandro R., Raimondo S. (2023). Exploiting the Opportunity to Use
Plant-Derived Nanoparticles
as Delivery Vehicles. Plants.

[86] Keivani
Nahr F., Ghanbarzadeh B., Hamishehkar H., Samadi Kafil H. (2018). Food Grade
Nanostructured Lipid Carrier for Cardamom Essential Oil: Preparation,
Characterization and Antimicrobial Activity. J. Funct.Foods.

[87] Sivalingam S., D J.
S. S., Golla G., Arunachalam L., Singh T., G K., A S., Malaichamy K. (2024). Encapsulation
of Essential Oil to Prepare Environment Friendly Nanobio-Fungicide
against Fusarium Oxysporum f.Sp. Lycopersici: An Experimental and
Molecular Dynamics Approach. Colloids Surf.,
A.

[88] Fuentes J. M., Jofré I., Tortella G., Benavides-Mendoza A., Diez M. C., Rubilar O., Fincheira P. (2024). The Mechanistic
Insights of Essential Oil of Mentha Piperita to Control Botrytis Cinerea
and the Prospection of Lipid Nanoparticles to Its Application. Microbiol. Res..

[89] Kaur M., Tandon R., Kalia A., Chander Mahajan B. V., Kairam N. (2024). ROS-Mediated Antifungal Activity
of Ocimum Essential
Oil-Loaded Nanoemulsions against Postharvest Fungal Pathogens of Kinnow. Food Biosci..

[90] Corkley I., Fraaije B., Hawkins N. (2022). Fungicide
Resistance Management:
Maximizing the Effective Life of Plant Protection Products. Plant Pathol..

[91] El-Baky N. A., Amara A. A. A. F. (2021). Recent Approaches towards Control of Fungal Diseases
in Plants: An Updated Review. JoF.

[92] Gómez-Lama
Cabanás C., Mercado-Blanco J. (2025). Groundbreaking Technologies and the
Biocontrol of Fungal Vascular Plant Pathogens. JoF.

[93] Ayaz M., Li C.-H., Ali Q., Zhao W., Chi Y.-K., Shafiq M., Ali F., Yu X.-Y., Yu Q., Zhao J.-T., Yu J.-W., Qi R.-D., Huang W.-K. (2023). Bacterial
and Fungal Biocontrol Agents for Plant Disease Protection: Journey
from Lab to Field, Current Status, Challenges, and Global Perspectives. Molecules.

[94] Rocha I. U., Bitencourt R. D. O. B., Freitas A. D. M., Moreira H. V. S., Magalhães K. L. D. A., Souza B. A. D., Golo P. S., Chaves D. S. D. A., Bittencourt V. R. E. P., Angelo I. D. C. (2024). Exploiting the Combination of Entomopathogenic
Fungi and Illicium Verum Essential Oil against Aedes Aegypti Larvae. Biol. Control.

[95] Si Z., Hou Z., Vikhe Y. S., Thappeta K. R. V., Marimuthu K., De P. P., Ng O. T., Li P., Zhu Y., Pethe K., Chan-Park M. B. (2021). Antimicrobial
Effect of a Novel Chitosan
Derivative and Its Synergistic Effect with Antibiotics. ACS Appl. Mater. Interfaces.

[96] Zarei M., Aminzadeh S., Zolgharnein H., Safahieh A., Daliri M., Noghabi K. A., Ghoroghi A., Motallebi A. (2011). Characterization
of a Chitinase with Antifungal Activity from a Native Serratia Marcescens
B4A. Braz. J. Microbiol..

[97] Nie H., Liao H., Wen J., Ling C., Zhang L., Xu F., Dong X. (2024). Foeniculum
Vulgare Essential Oil Nanoemulsion Inhibits
Fusarium Oxysporum Causing Panax Notoginseng Root-Rot Disease. J. Ginseng Res..

[98] Yeasmin S., Dipto A. R., Zakir A. B., Shovan S. D., Suvo M. A. H., Bhuiyan M. A., Amin Md. N., Rashid T. U., Islam S., Habib A. (2024). Nanopriming and AI for Sustainable
Agriculture: Boosting Seed Germination
and Seedling Growth with Engineered Nanomaterials, and Smart Monitoring
through Deep Learning. ACS Appl. Nano Mater..

[99] Bhaskara
Reddy M. V., Arul J., Angers P., Couture L. (1999). Chitosan Treatment
of Wheat Seeds Induces Resistance to *Fusarium Graminearum* and Improves Seed Quality. J. Agric. Food
Chem..

[100] Bondareva L., Fedorova N. (2021). Pesticides:
Behavior in Agricultural
Soil and Plants. Molecules.

[101] Sapna, Sharma C., Pathak P., Yadav S. P., Gautam S. (2024). Potential
of Emerging
“All-Natural” Edible Coatings to Prevent Post-Harvest
Losses of Vegetables and Fruits for Sustainable Agriculture. Prog. Org. Coat..

[102] Yadav N., Garg V. K., Chhillar A. K., Rana J. S. (2023). Recent
Advances in Nanotechnology for the Improvement of Conventional Agricultural
Systems: A Review. Plant Nano Biol..

[103] Shelar A., Nile S. H., Singh A. V., Rothenstein D., Bill J., Xiao J., Chaskar M., Kai G., Patil R. (2023). Recent Advances in Nano-Enabled Seed Treatment Strategies for Sustainable
Agriculture: Challenges, Risk Assessment, and Future Perspectives. Nano-Micro Lett..

[104] Afzal I., Javed T., Amirkhani M., Taylor A. G. (2020). Modern Seed Technology: Seed Coating Delivery Systems
for Enhancing Seed and Crop Performance. Agriculture.

[105] Riseh R. S., Vazvani M. G., Vatankhah M., Kennedy J. F. (2024). Chitosan Coating of Seeds Improves the Germination
and Growth Performance of Plants: A Rreview. Int. J. Biol. Macromol..

[106] Cardarelli M., Woo S. L., Rouphael Y., Colla G. (2022). Seed Treatments
with Microorganisms Can Have a Biostimulant Effect by Influencing
Germination and Seedling Growth of Crops. Plants.

[107] Guedes N. A., Peccini L. R., Bigui W. C. C., Baute J. L., Feu K. S., Chaves K. F., De Oliveira Pires R. M., Villanova J. C. O., Morais P. A. B., Costa A. V., De Queiroz V. T. (2025). Thyme Essential
Oil-Loaded Emulsion as a Potential Nanofungicide for Controlling Fusarium
Oxysporum f. Sp. Lycopersici. Eur. J. Plant
Pathol..

[108] Alim M. A., Hossain S. I., Ditta A., Hasan M. K., Islam M. R., Hafeez A. S. M. G., Khan M. A. H., Chowdhury M. K., Pramanik M. H., Al-Ashkar I., El Sabagh A., Islam M. S. (2023). Comparative Efficacy of Foliar Plus
Soil Application
of Urea versus Conventional Application Methods for Enhanced Growth,
Yield, Agronomic Efficiency, and Economic Benefits in Rice. ACS Omega.

[109] Hong J., Wang C., Wagner D. C., Gardea-Torresdey J. L., He F., Rico C. M. (2021). Foliar Application of Nanoparticles: Mechanisms of
Absorption, Transfer, and Multiple Impacts. Environ. Sci.: Nano.

[110] Avellan A., Yun J., Morais B. P., Clement E. T., Rodrigues S. M., Lowry G. V. (2021). Critical Review: Role of Inorganic
Nanoparticle Properties on Their Foliar Uptake and *in Planta* Translocation. Environ. Sci. Technol..

[111] Nguyen M.-H., Tran T.-N.-M., Vu N.-B.-D. (2022). Antifungal
Activity
of Essential Oil-Encapsulated Lipid Nanoemulsions Formulations against
Leaf Spot Disease on Tomato Caused by *Alternaria Alternata*. Arch. Phytopathol. Plant Protect..

[112] Choudhary R. C., Kumaraswamy R. V., Kumari S., Sharma S. S., Pal A., Raliya R., Biswas P., Saharan V. (2019). Zinc Encapsulated Chitosan
Nanoparticle to Promote Maize Crop Yield. Int.
J. Biol. Macromol..

[113] Chen S., Guo X., Zhang B., Nie D., Rao W., Zhang D., Lü J., Guan X., Chen Z., Pan X. (2023). Mesoporous Silica Nanoparticles
Induce Intracellular Peroxidation
Damage of *Phytophthora Infestans*: A New Type of Green
Fungicide for Late Blight Control. Environ.
Sci. Technol..

[114] Daniel P. E., Bedmar F., Costa J. L., Aparicio V. C. (2002). Atrazine
and Metribuzin Sorption in Soils of the Argentinean Humid Pampas. Environ. Toxicol. Chem..

[115] Celano G., Šmejkalová D., Spaccini R., Piccolo A. (2008). Interactions of Three S-Triazines with Humic Acids
of Different Structure. J. Agric. Food Chem..

[116] Martinazzo R., Dick D. P., Hirsch M. M., Leite S. B., Peralba M. D. C. R. (2011). Sorção de Atrazina
e de Mesotriona Em
Latossolos e Estimativa Do Potencial de Contaminação. Quím. Nova.

[117] Chen H. (2018). Metal Based Nanoparticles in Agricultural
System: Behavior, Transport,
and Interaction with Plants. Chem. Speciat.
Bioavailab..

[118] Wang X., Xie H., Wang P., Yin H. (2023). Nanoparticles
in Plants: Uptake, Transport and Physiological Activity in Leaf and
Root. Materials.

[119] Zhao L., Peralta-Videa J. R., Ren M., Varela-Ramirez A., Li C., Hernandez-Viezcas J. A., Aguilera R. J., Gardea-Torresdey J. L. (2012). Transport
of Zn in a Sandy Loam Soil Treated with ZnO NPs and Uptake by Corn
Plants: Electron Microprobe and Confocal Microscopy Studies. Chem. Eng. J..

[120] Judy J. D., Unrine J. M., Rao W., Wirick S., Bertsch P. M. (2012). Bioavailability of Gold Nanomaterials
to Plants: Importance
of Particle Size and Surface Coating. Environ.
Sci. Technol..

[121] Tripathi D. K., Shweta, Singh S., Singh S., Singh S., Pandey R., Singh V. P., Sharma N. C., Prasad S. M., Dubey N. K., Chauhan D. K. (2017). An Overview on Manufactured
Nanoparticles
in Plants: Uptake, Translocation, Accumulation and Phytotoxicity. Plant Physiol. Biochem..

[122] Lv J., Christie P., Zhang S. (2019). Uptake, Translocation,
and Transformation
of Metal-Based Nanoparticles in Plants: Recent Advances and Methodological
Challenges. Environ. Sci.: Nano.

[123] Bueno V., Wang P., Harrisson O., Bayen S., Ghoshal S. (2022). Impacts of a Porous Hollow Silica
Nanoparticle-Encapsulated Pesticide Applied to Soils on Plant Growth
and Soil Microbial Community. Environ. Sci.:
Nano.

[124] Silva E. F. D., Santos F. A. L. D., Pires H. M., Bastos L. M., Ribeiro L. N. D. M. (2025). Lipid Nanoparticles
Carrying Essential Oils for Multiple
Applications as Antimicrobials. Pharmaceutics.

[125] Bisht, S. Nanopesticide Market by Product Type, 2024. https://www.credenceresearch.com/report/nanopesticide-market (accessed 2025–01–10).

[126] Ke M., Zhang K., Hicks A. L., Wu F., You J. (2025). A Life Cycle
Risk Assessment of Nanopesticides in Freshwater. Environ. Sci. Ecotechnology.

[127] Burandt Q. C., Deising H. B., Von Tiedemann A. (2024). Further Limitations
of Synthetic Fungicide Use and Expansion of Organic Agriculture in
Europe Will Increase the Environmental and Health Risks of Chemical
Crop Protection Caused by Copper-Containing Fungicides. Environ. Toxicol. Chem..

[128] Kah M., Kookana R. S., Gogos A., Bucheli T. D. (2018). A Critical Evaluation
of Nanopesticides and Nanofertilizers against Their Conventional Analogues. Nat. Nanotechnol..

[129] Xuan L., Ju Z., Skonieczna M., Zhou P., Huang R. (2023). Nanoparticles-induced Potential Toxicity
on Human Health: Applications, Toxicity Mechanisms, and Evaluation
Models. MedComm.

[130] Grillo R., Fraceto L. F., Amorim M. J. B., Scott-Fordsmand J. J., Schoonjans R., Chaudhry Q. (2021). Ecotoxicological and Regulatory Aspects
of Environmental Sustainability of Nanopesticides. J. Hazard. Mater..

[131] Gómez-Llorente H., Hervás P., Pérez-Esteve E. ´., Barat J. M., Fernández-Segovia I. (2022). Nanotechnology
in the Agri-Food Sector: Consumer Perceptions. NanoImpact.

[132] Boholm Å., Larsson S. (2019). What Is the Problem?
A Literature
Review on Challenges Facing the Communication of Nanotechnology to
the Public. J. Nanopart. Res..

[133] Morgado R. G., Pavlaki M. D., Soares A. M. V. M., Loureiro S. (2022). Terrestrial Organisms React. Differently to Nano and
Non-Nano Cu­(OH)­2 Forms. Sci. Total Environ..

[134] Eghbalinejad M., Hofman J., Kotouček J., Grillo R., Hochmanová Bílková Z., Reiff N., Höss S. (2024). Nano-Enabled Pesticides: A Comprehensive
Toxicity Assessment of Tebuconazole Nanoformulations with Nematodes
at Single Species and Community Level. Environ.
Sci. Eur..

[135] Banu A. N., Kudesia N., Raut A. M., Pakrudheen I., Wahengbam J. (2021). Toxicity, Bioaccumulation, and Transformation
of Silver
Nanoparticles in Aqua Biota: A Review. Environ.
Chem. Lett..

[136] Ghosh, M. ; Kumar, R. Regulatory Issues in Nanotechnology. In Nanotechnology Theranostics in Livestock Diseases and Management; Prasad, M. , Kumar, R. , Ghosh, M. , Syed, S. M. , Chakravarti, S. , Eds.; Livestock Diseases and Management; Springer Nature Singapore: Singapore, 2024; pp 765–788.

[137] Ali F., Neha K., Parveen S. (2023). Current Regulatory
Landscape of Nanomaterials
and Nanomedicines: A Global Perspective. J.
Drug Delivery Sci. Technol..

[138] Rana L., Kumar M., Rajput J., Kumar N., Sow S., Kumar S., Kumar A., Singh S. N., Jha C. K., Singh A. K., Ranjan S., Sahoo R., Samanta D., Nath D., Panday R., Raigar B. L. (2024). Nexus between Nanotechnology
and Agricultural Production Systems: Challenges and Future Prospects. Discov. Appl. Sci..

[139] Khairnar S. V., Pagare P., Thakre A., Nambiar A. R., Junnuthula V., Abraham M. C., Kolimi P., Nyavanandi D., Dyawanapelly S. (2022). Review on the Scale-Up Methods for
the Preparation
of Solid Lipid Nanoparticles. Pharmaceutics.

[140] Mehta M., Bui T. A., Yang X., Aksoy Y., Goldys E. M., Deng W. (2023). Lipid-Based Nanoparticles
for Drug/Gene
Delivery: An Overview of the Production Techniques and Difficulties
Encountered in Their Industrial Development. ACS Mater. Au.

[141] Alfutaimani A. S., Alharbi N. K., Alahmari A., Alqabbani A., Aldayel A. M. (2024). Exploring the Landscape of Lipid
Nanoparticles (LNPs):
A Comprehensive Review of LNPs Types and Biological Sources of Lipids. Int. J. Pharm.: X.

[142] Zhou Y., Ge Q., Wang X., Wang Y., Sun Q., Wang J., Yang T., Wang C. (2025). Advances in Lipid Nanoparticle-Based
Disease Treatment. ChemMedChem.

[143] Lüdtke F. L., Silva T. J., Da Silva M. G., Hashimoto J. C., Ribeiro A. P. B. (2025). Lipid Nanoparticles: Formulation,
Production Methods
and Characterization Protocols. Foods.

[144] Emami F., Keihan Shokooh M., Mostafavi Yazdi S. J. (2023). Recent
Progress in Drying Technologies for Improving the Stability and Delivery
Efficiency of Biopharmaceuticals. J. Pharm.
Invest..

[145] Santonocito D., Sarpietro M. G., Castelli F., Lauro M. R., Torrisi C., Russo S., Puglia C. (2023). Development of Solid
Lipid Nanoparticles as Dry Powder: Characterization and Formulation
Considerations. Molecules.

[146] Ball R., Bajaj P., Whitehead K. (2017). Achieving
Long-Term Stability of Lipid Nanoparticles: Examining the Effect of
pH, Temperature, and Lyophilization. Int. J.
Nanomed..

[147] Soleimanian Y., Goli S. A. H., Varshosaz J., Maestrelli F. (2019). Β-sitosterol Lipid Nano Carrier Based on Propolis
Wax and Pomegranate Seed Oil: Effect of Thermal Processing, pH, and
Ionic Strength on Stability and Structure. Eur.
J. Lipid Sci. Technol..

[148] Liu Z., Okeke C. I., Zhang L., Zhao H., Li J., Aggrey M. O., Li N., Guo X., Pang X., Fan L., Guo L. (2014). Mixed Polyethylene Glycol-Modified Breviscapine-Loaded
Solid Lipid Nanoparticles for Improved Brain Bioavailability: Preparation,
Characterization, and In Vivo Cerebral Microdialysis Evaluation in
Adult Sprague Dawley Rats. AAPS PharmSciTech.

[149] Pandey R., Khuller G. K. (2005). Solid Lipid Particle-Based
Inhalable
Sustained Drug Delivery System against Experimental Tuberculosis. Tuberculosis.

[150] Rabelo R. S., Oliveira I. F., Da Silva V. M., Prata A. S., Hubinger M. D. (2018). Chitosan Coated Nanostructured Lipid Carriers (NLCs)
for Loading Vitamin D: A Physical Stability Study. Int. J. Biol. Macromol..

[151] Malekmohammadi M., Ghanbarzadeh B., Hanifian S., Samadi Kafil H., Gharekhani M., Falcone P. M. (2023). The Gelatin-Coated Nanostructured
Lipid Carrier (NLC) Containing Salvia Officinalis Extract: Optimization
by Combined D-Optimal Design and Its Application to Improve the Quality
Parameters of Beef Burger. Foods.

[152] Khanna K., Kohli S. K., Handa N., Kaur H., Ohri P., Bhardwaj R., Yousaf B., Rinklebe J., Ahmad P. (2021). Enthralling the Impact of Engineered Nanoparticles on Soil Microbiome:
A Concentric Approach towards Environmental Risks and Cogitation. Ecotoxicol. Environ. Saf..

[153] Khan S. T., Adil S. F., Shaik M. R., Alkhathlan H. Z., Khan M., Khan M. (2022). Engineered Nanomaterials
in Soil:
Their Impact on Soil Microbiome and Plant Health. Plants.

[154] Kapeleka J. A., Mwema M. F. (2024). State of Nano Pesticides
Application
in Smallholder Agriculture Production Systems: Human and Environmental
Exposure Risk Perspectives. Heliyon.

[155] Espiña, B. ; Rodriguez-Lorenzo, L. Environmental Nanosafety. In Nanosafety; Alfaro-Moreno, E. , Murphy, F. , Eds.; Springer Nature Switzerland: Cham, 2025; pp 403–437.

[156] Hofmann T., Lowry G. V., Ghoshal S., Tufenkji N., Brambilla D., Dutcher J. R., Gilbertson L. M., Giraldo J. P., Kinsella J. M., Landry M. P., Lovell W., Naccache R., Paret M., Pedersen J. A., Unrine J. M., White J. C., Wilkinson K. J. (2020). Technology Readiness and Overcoming
Barriers to Sustainably Implement Nanotechnology-Enabled Plant Agriculture. Nat. Food.

[157] Jassim A. Y., Karnwal A., Pant G., Gaur A., Selvaraj M., Dutta J., Sachan R. S. K., Kumar M., Nesterova N. (2025). Harnessing Nanotechnology for Abiotic Stress Mitigation
in Agriculture: A Sustainable Approach to Crop Resilience. J. Plant Interact..

[158] Pirouzifard M. K., Hamishehkar H., Pirsa S. (2020). Cocoa Butter and Cocoa
Butter Substitute as a Lipid Carrier of *Cuminum cyminum* L. Essential Oil; Physicochemical Properties, Physical Stability
and Controlled Release Study. J. Mol. Liq..

